# Supplementary material for: A human liver cell-based system modeling a clinical prognostic liver signature for therapeutic discovery
Source: Nat Commun. 2021 Sep 17;12:5525. doi: 10.1038/s41467-021-25468-9 (PMC8448834; doi:10.1038/s41467-021-25468-9)
Supplement: Supplementary file 1 — Supplementary Information [file 41467_2021_25468_MOESM1_ESM.pdf]

# A human liver cell-based system modeling a clinical prognostic liver signature for therapeutic discovery

Emilie Crouchet[1,2], Simonetta Bandiera[1,2], Naoto Fujiwara[3], Shen Li[4], Hussein El Saghire[1,2], Mirian Fernández-Vaquero[5,6], Tobias Riedl[5,6], Xiaochen Sun[3], Hadassa Hirschfield[3], Frank Jühling[1,2,], Shijia Zhu[3], Natascha Roehlen[1,2], Clara Ponsolles[1,2], Laura Heydmann[1,2], Antonio Saviano[1,2,7], Tongqi Qian[3], Anu Venkatesh[3], Joachim Lupberger[1,2], Eloi R. Verrier[1,2], Mozhdeh Sojoodi[4], Marine A Oudot[1,2], François H.T. Duong[1,2,8], Ricard Masia[9], Lan Wei[4], Christine Thumann[1,2], Sarah C. Durand[1,2], Victor González-Motos[1,2], Danijela Heide[5], Jenny Hetzer[5], Shigeki Nakagawa[3], Atsushi Ono[10], Won-Min Song[11], Takaaki Higashi[12], Roberto Sanchez[13], Rosa S. Kim[14], C. Billie Bian[11], Karun Kiani[15,16], Tom Croonenborghs[15,16,17], Aravind Subramanian[15], Raymond T. Chung[18], Beate K. Straub[19], Detlef Schuppan[20,21], Maliki Ankavay[22], Laurence Cocquerel[22], Evelyne Schaeffer[23], Nicolas Goossens[24], Anna P. Koh[3], Milind Mahajan[11], Venugopalan D. Nair[25], Ganesh Gunasekaran[26], Myron E. Schwartz[26], Nabeel Bardeesy[27], Alex K. Shalek[18,28,29], Orit Rozenblatt-Rosen[16]†, Aviv Regev[16,30]†, Emanuele Felli[1,2,7], Patrick Pessaux[1,2,7], Kenneth K. Tanabe[4], Mathias Heikenwälder[5], Catherine Schuster[1,2,], Nathalie Pochet[15,16], Mirjam B. Zeisel[1,2]†, Bryan C. Fuchs[4*]†, Yujin Hoshida[3*], Thomas F. Baumert[1,2,7*]

*** corresponding authors**

## Supplementary Figures

## Supplementary Figure 1

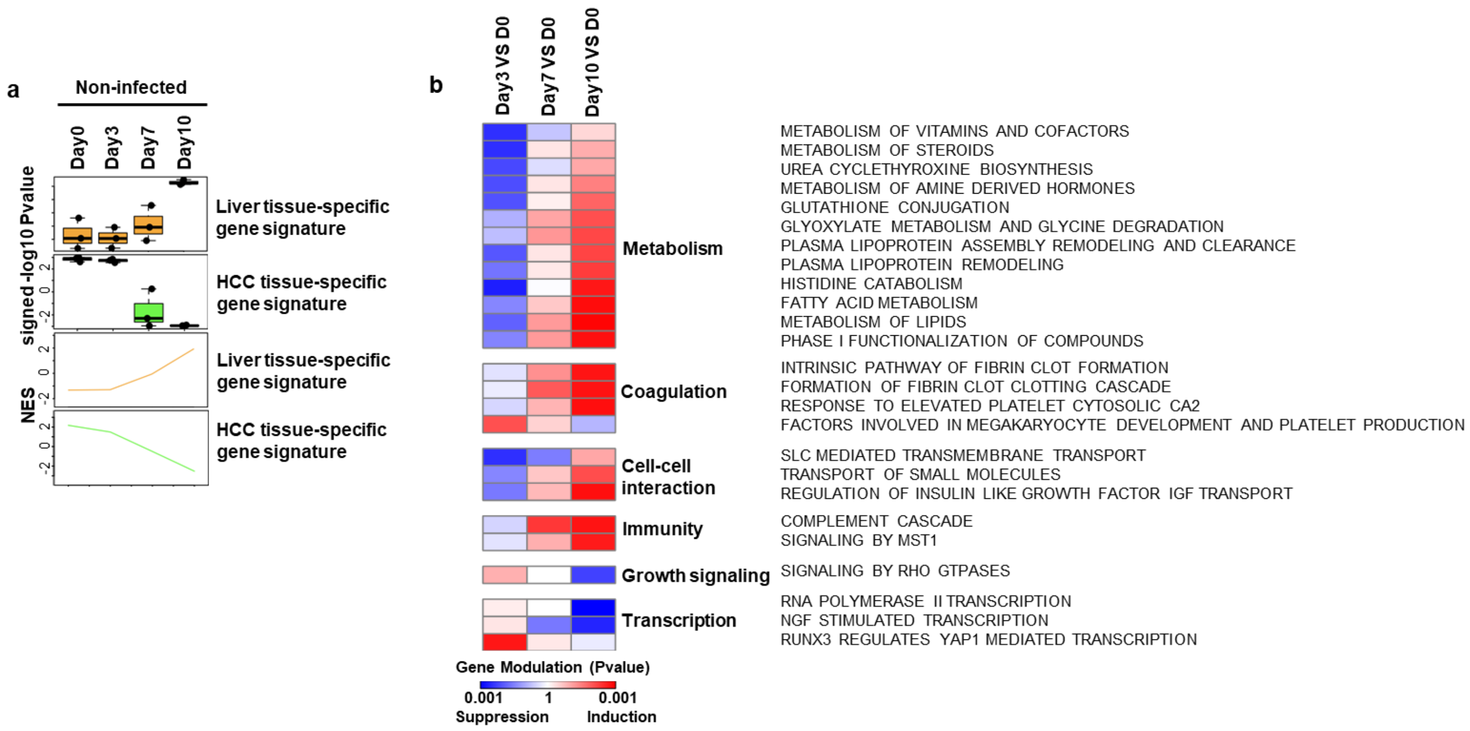


**Supplementary Figure 1 (related to Figure 1): Time-course of molecular pathway modulation during DMSO-induced differentiation of Huh7.5.1 into hepatocyte-like cells. a.** DMSO-differentiation of Huh7.5.1 (Huh7.5.1$^{dif}$) cells induced a hepatocyte-like phenotype a gradual shift of a liver cancer to a non-cancer liver tissue-specific gene signature over 10 days. Modulation of 714 genes specifically over-expressed in human non-malignant liver tissue (liver tissue-specific gene signature) and 874 genes specifically over-expressed in liver cancer tissues (liver cancer tissue-specific gene signature) defined in genome-wide transcriptome profiles of 200 clinical specimens. In box and whisker plots, boxes represent the 75th and 25th percentiles, the whiskers represent the most extreme data points within interquartile range × 1.5, and the horizontal bar represents the median. Circle indicates actual observation for each sample. NES: normalized enrichment score. Results are from one experiment performed in triplicate. **b.** Induction or suppression of molecular pathways was assessed on the gene expression fold change of Day 3-10 with Day 0 (without DMSO) as internal control, using GSEA based on a comprehensive curated pathway database (Reactome: www.reactome.org). Significance of induction or suppression of each pathway is shown by *p* values of gene set enrichment. Gradually induced or suppressed gene sets overtime were selected based on absolute Spearman correlation coefficient with a vector (1, 2, 3) = 1 and at least one $p < 0.01$ among three time points. Results are representative of one experiment performed in triplicate. Source data are provided as a Source Data file.

## Supplementary Figure 2

**a. Functional categories in 186-gene signature**

| Poor-prognosis genes (73 genes) | Good-prognosis genes (113 genes) |
|---|---|
| Interferon pathway | Bile acid biosynthesis |
| IL-6 pathway | Glycerolipid metabolism |
| TNF-α pathway | Fatty acid metabolism |
| NF-κB pathway | Androgen/estrogen metabolism |
| TGF-β pathway κ | Tryptophan metabolism |
| Activated hepatic stellate cells | Valine/leucine/isoleucine metabolism |
| | Inositol phosphate metabolism |
| | Butanoate metabolism |
| | Beta alanine metabolism |
| | Pyruvate metabolism |
| | Glycine serine/threonine metabolism |
| | DNA damage repair |

**b. Cell-based system: 186 gene signature**

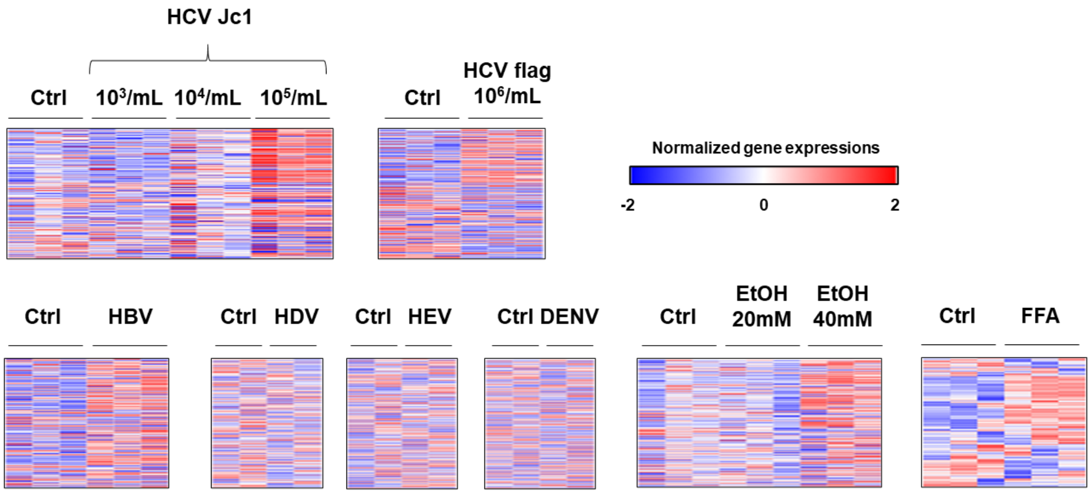


**Supplementary Figure 2 (related to Figure 1): Functional categories in 186-gene signature and detailed PLS gene expression profiles of key analyses. a.** The gene signature consists in 73 poor-prognosis genes (mainly involved in inflammation and immune response) and in 113 good-prognosis genes (mainly involved in normal liver function and metabolism). For a complete gene list, see[1]. **b** Detailed PLS gene expression profiles of Huh7.5.1$^{dif}$ cells (cell-based system) for the 186 gene signature, under various experimental interventions shown in Fig1d, 1g and 1h. Heatmaps show the mean expression of the 186 gene signature (z scores of log2 normalized data). Gene expression was normalized according to 6 different housekeeping genes. Source data are available in GEO database GSE66843. The full PLS gene list is detailed in Supplementary Data 1.

## Supplementary Figure 3

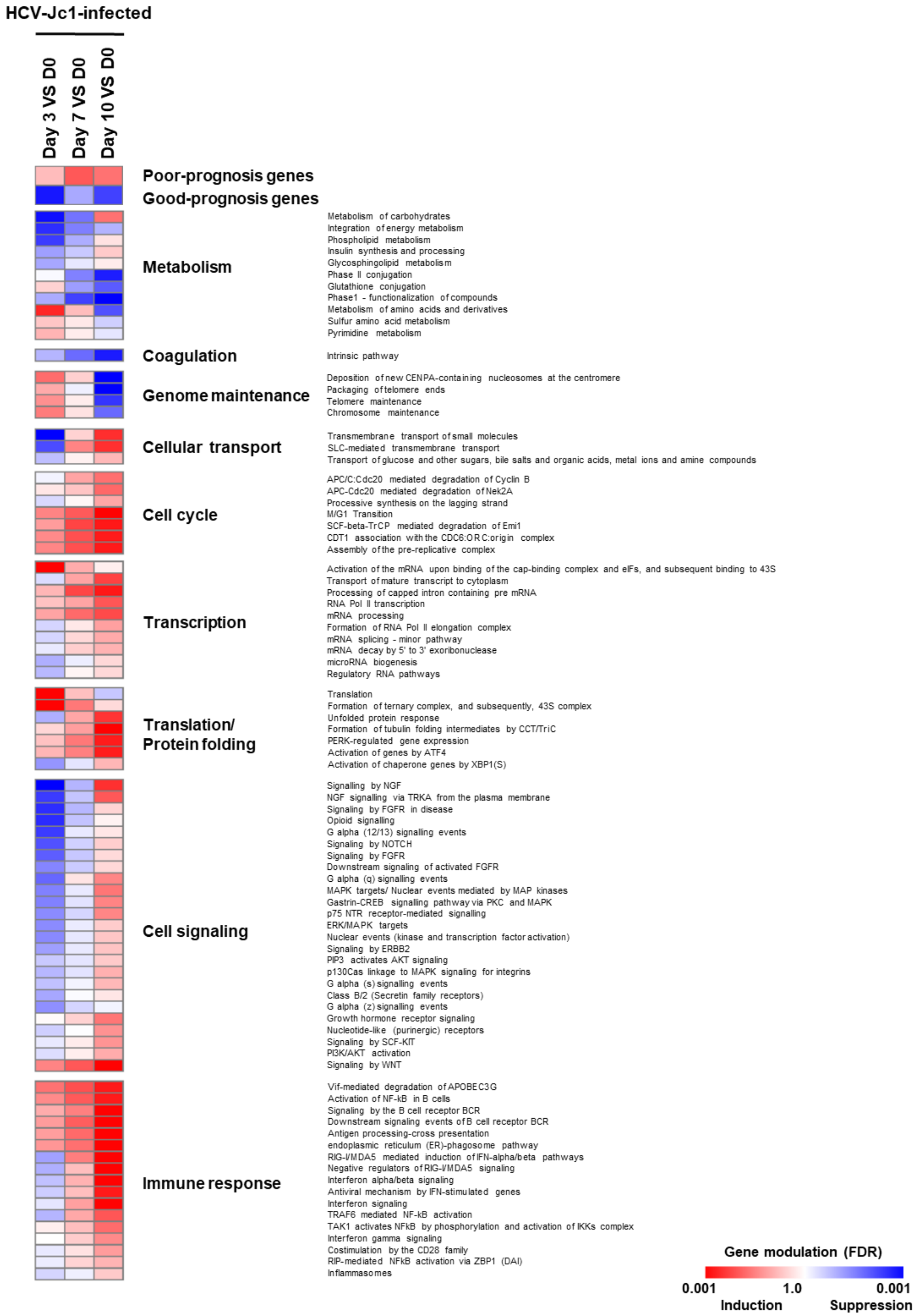

**Supplementary Figure 3 (related to Figure 1): Time-course of molecular pathway modulation by persistent HCV infection (HCV Jc1) versus mock-infected control cells (D0) in Huh7.5.1$^{dif}$ cells.** Huh7.5.1$^{dif}$ cells were infected with HCV Jc1, and RNA was isolated and analyzed as described in Fig. 1a. Induction or suppression of molecular pathways was assessed using GSEA on a comprehensive curated pathway database (Reactome (www.reactome.org)) using non-infected mock cells as a control (D0). Significance of induction or suppression of each pathway is shown by false discovery rate (FDR) of gene set enrichment. In the heatmap, dense red indicates significant induction, blue indicates significant suppression. Gradually induced or suppressed gene sets overtime were selected based on absolute correlation coefficient with a vector (1, 2, 3) > 0.95 and maximum index – minimum index > 1. Results are from one experiment performed in triplicate. Metabolism- and coagulation-related pathways, except carbohydrate metabolism pathway, were suppressed by HCV Jc1 infection overtime. Genome maintenance-related pathways were also suppressed. Cell proliferation-, signaling-, and immune response-related pathways were gradually induced. Source data are provided as a Source Data file.

**Supplementary Figure 4**

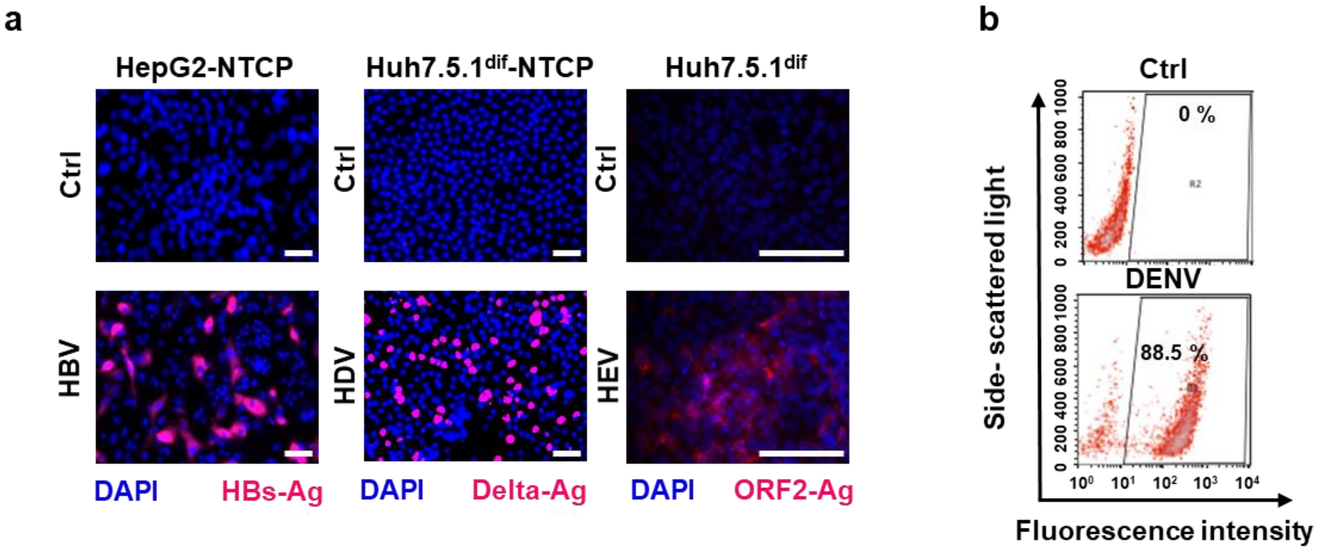


**Supplementary Figure 4 (related to Figure 1). Cell-based models were infected with different viruses or exposed to metabolic cues. a.** Confirmation of viral infection by immunodetection of HBV, HDV and HEV viral antigens of the experiment shown in Fig. 1g. Scale bar: 50 µm. **b.** Flow cytometry analysis of DENV infection in Huh7.5.1$^{dif}$ cells at 24 h post-infection (intracellular content of DENV-E protein).

# Supplementary Figure 5

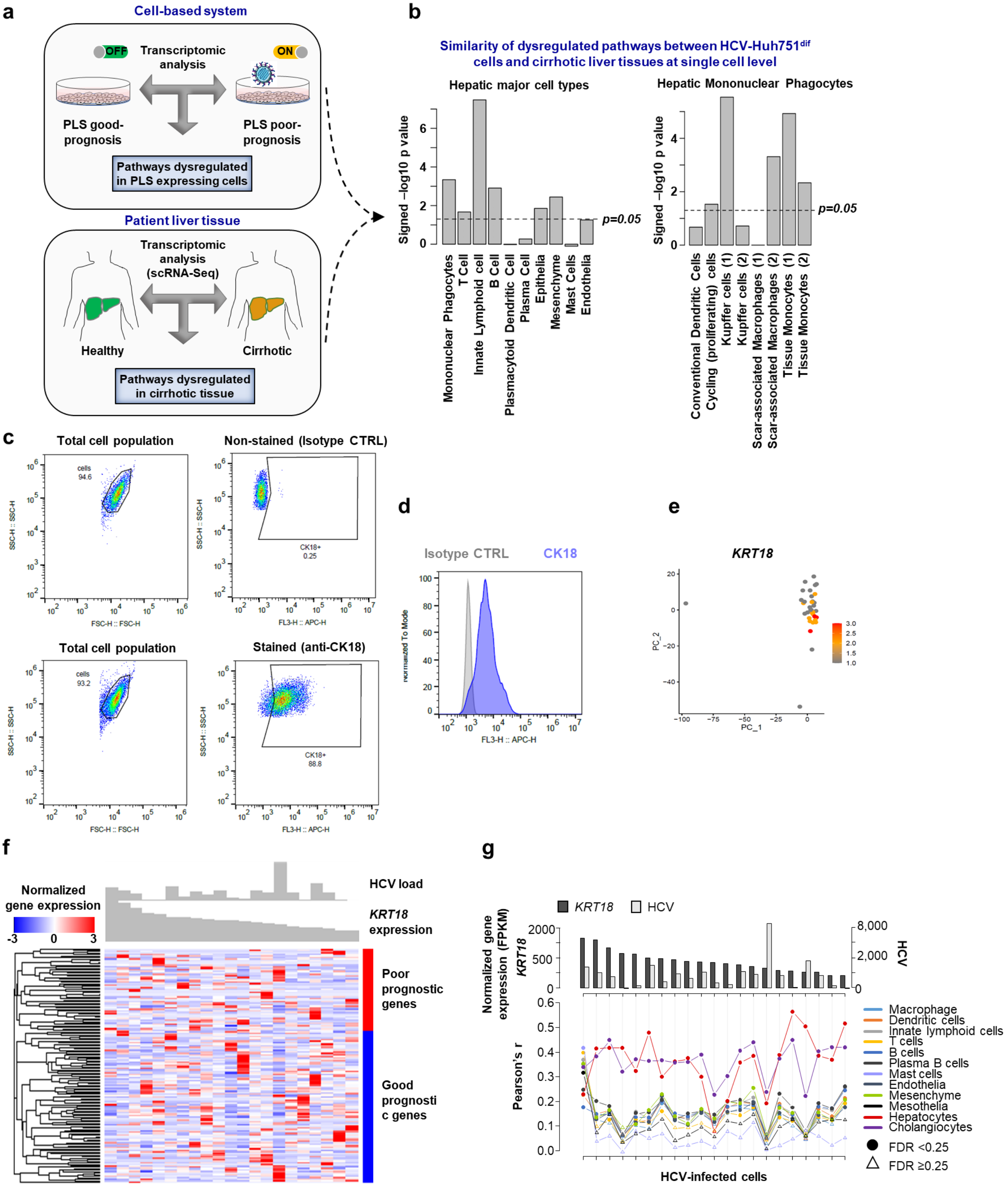

**Supplementary Figure 5 (related to Figure 1). The cPLS system captures shared molecular pathways between epithelial cells and NPCs. a.** Experimental approach. **b.** Similarity of global transcriptome between the cPLS model described in Fig. 1 and single-cell transcriptome profiles of liver cells (including macrophages and endothelial cells) isolated from human cirrhotic livers[2]. See Method. Spearman correlation test. Significance threshold: false discovery rate (FDR) < 0.05. **c-e.** Heterogeneity of cPLS monoculture model assessed by flow cytometry and scRNA-Seq analysis. (c) Gating strategy. Gating was based on FCS area vs SSC area to select the starting cell population (log scale, 10,000 cells analyzed). For detection of CK18, a marker of hepatocyte, gating was established based on cells incubated with non-specific isotype control (CTRL) (d) CK18 expression assessed by flow cytometry in Huh7.5.1$^{dif}$ cells. Histogram shows distribution of CK18 expression. (e) t.SNE map showing *KRT18* (encoding for CK18) expression in Huh7.5.1$^{dif}$ cells at the single cell level shown in Fig. 1e. **f.** PLS gene expression in HCV-infected Huh7.5.1$^{dif}$ cells (n = 21) sorted by *KRT18* expression level. The HCV viral load is shown for each cell. **g.** Similarity of HCV-infected Huh7.5.1$^{dif}$ cells (n = 21) with different liver cell compartment isolated from human cirrhotic livers[2]. *X* axis represents HCV-infected cells and *y* axis represents Pearson's *r* with mean gene expression levels of PLS genes in each liver cell type. HCV-infected cells are sorted by *KRT18* expression level. FDR = false discovery rate. Source data are provided as a Source Data file.

**Supplementary Figure 6**

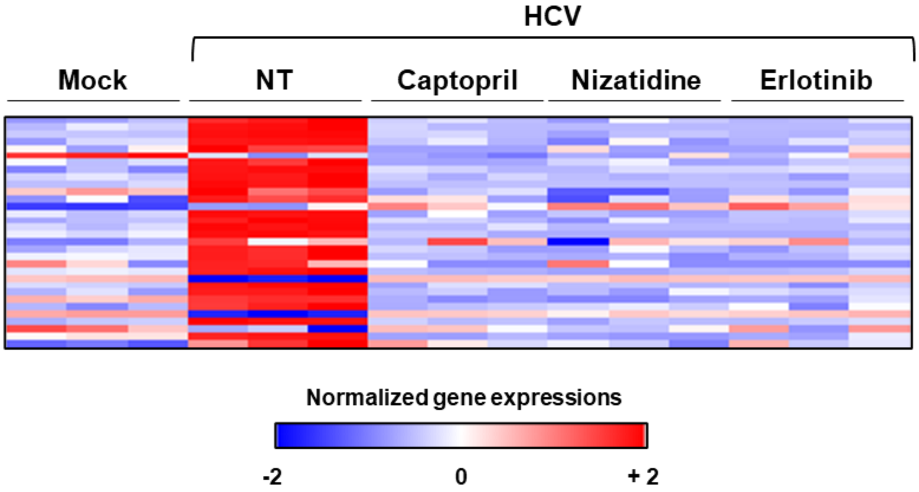


**Supplementary Figure 6 (related to Figure 2a). PLS gene expression of top scoring compounds presented as normalized expression.** Detailed PLS 32 gene expression profiles of Huh7.5.1$^{dif}$ cells (cell-based system) CTRL (Mock), HCV-infected non-treated (NT) and HCV-infected treated with the 2 best hits (captopril and nizatidine) as well as with the control for PLS reversion, erlotinib, as shown in Fig. 2. Heatmaps show the mean expression of the 32 gene signature (z scores of log2 normalized data). Gene expression was normalized according to 6 different housekeeping genes. Source data are provided as a Source Data file.

**Supplementary Figure 7**

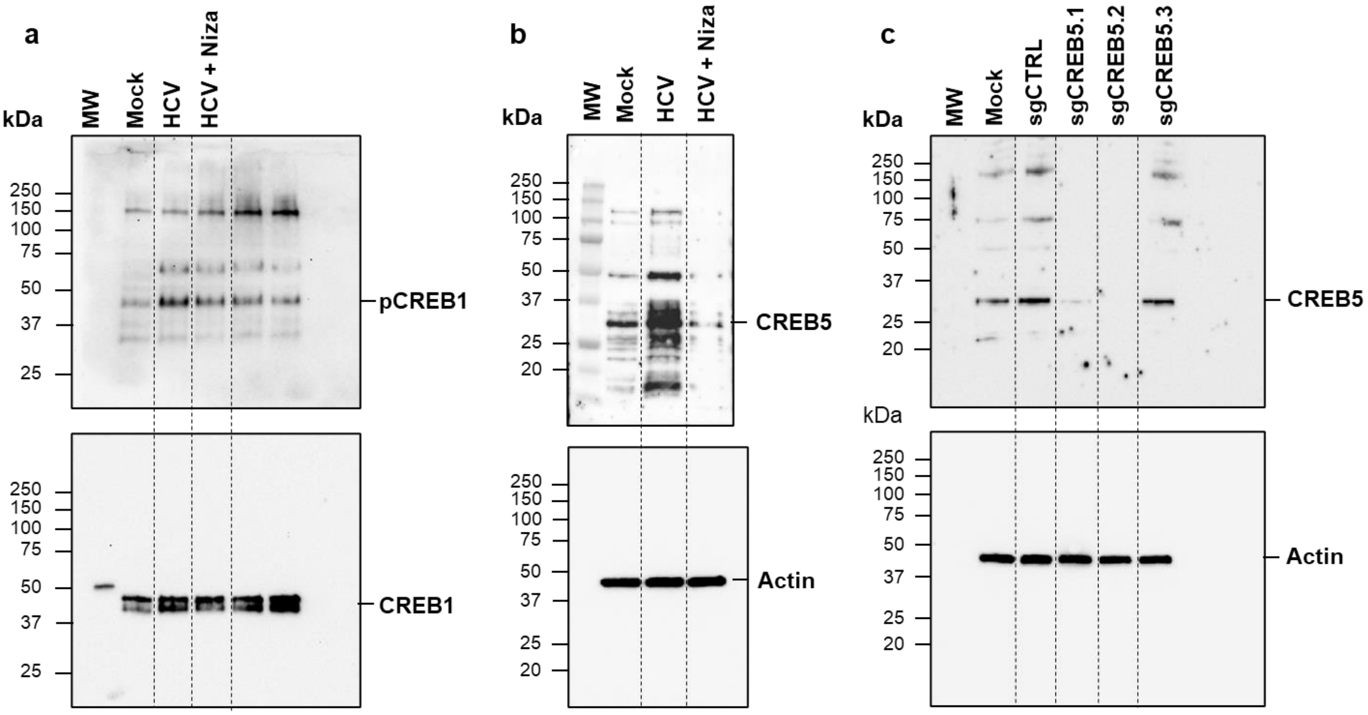


**Supplementary Figure 7: Full length immunoblots from Figure 3.**

To demonstrate the specificity of the applied antibodies, full-length gels are shown for **(a and b)** Fig. 3e and **(c)** Fig. 3f. Target protein names indicate the bands shown in the Fig. 3. Protein analysis was performed in cell lysates from the same experiment using a reducing 12% SDS-PAGE gel electrophoresis. PVDF membranes were probed for pCREB1 and total CREB1 (rabbit polyclonal Ab, Abcam, 1:1,000), CREB5 (mAb Ab, Novus, 1:1,000) and for Actin, the loading control (mouse mAb, Abcam, 1:2,000). Secondary antibodies were horseradish peroxidase-conjugated goat anti-rabbit antibody (Jackson Immunoreasearch, 1:10,000) for pCREB1 and CREB1 and horseradish peroxidase-conjugated sheep anti-mouse antibody (GE Healthcare, 1:5,000) for CREB5 and Actin. The marker sizes (Precision Plus Protein Standards All Blue, BioRad) are indicated. Contrasts of western blotting images were equally adjusted for entire membranes using Bio-Rad image analysis software. Due to similar size of the proteins, analysis of pCREB1, total CREB1, CREB5 and Actin was performed on different gels however using the same cell lysates from the same experiment. MW = molecular weight markers. References of the antibodies are provided in Supplementary Table 4. Source data are provided as a Source Data file.

## Supplementary Figure 8

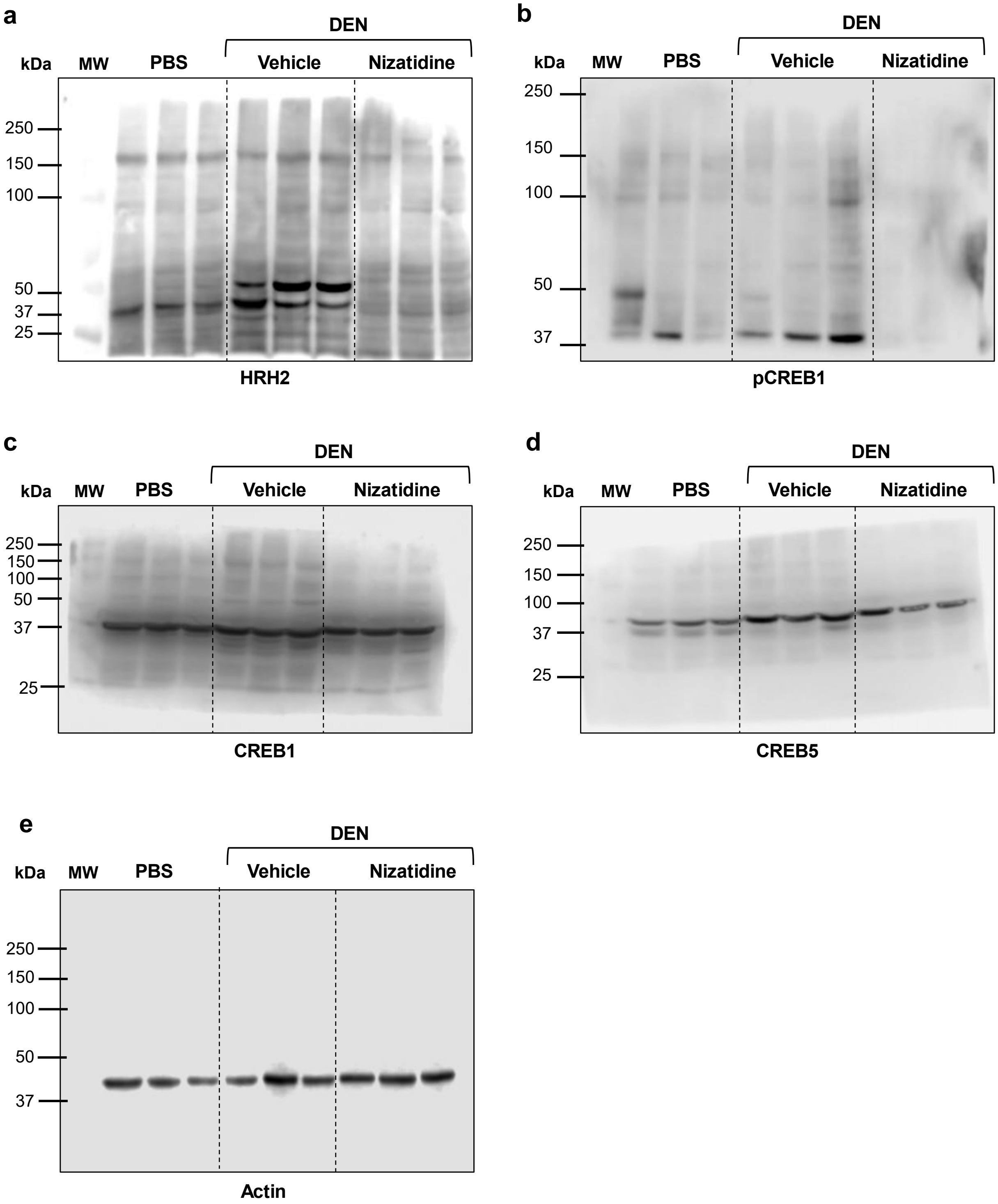

**Supplementary Figure 8 (related to Figure 4): Full length immunoblots from the DEN cirrhosis rat model.** To demonstrate the specificity of the applied antibodies, full-length gels are shown for Fig. 4k.Target protein names indicate the bands shown in the Fig. 4k. Protein analysis was performed in liver lysates from the same experiment using a reducing 12% SDS-PAGE gel electrophoresis. PVDF membranes were probed for HRH2 (rabbit polyclonal Ab, 1:1,000), pCREB1 and total CREB1 (rabbit monoclonal Ab, Cell Signaling, 1:1,000), CREB5 (mAb Ab, LS Bioscience, 1:1,000) and for Actin (mouse mAb, Abcam, 1:5,000). Due to the close molecular weight of the target proteins, analyses were performed on independent PVDF membranes done in the same conditions and using the same lysates. Secondary antibodies were horseradish peroxidase-conjugated goat anti-rabbit antibody (Cell Signaling, 1:5,000) for HRH2, pCREB1 and CREB1 and horseradish peroxidase-conjugated goat anti-mouse antibody (Thermofisher Scientific, 1:5,000) for CREB5 and Actin. The marker sizes (Precision Plus Protein Standards All Blue, BioRad) are indicated. Contrasts of western blotting images were equally adjusted for entire membranes using Bio-Rad image analysis software. MW = molecular weight markers. References of the antibodies are provided in Supplementary Table 4. Source data are provided as a Source Data file.

**Supplementary Figure 9**

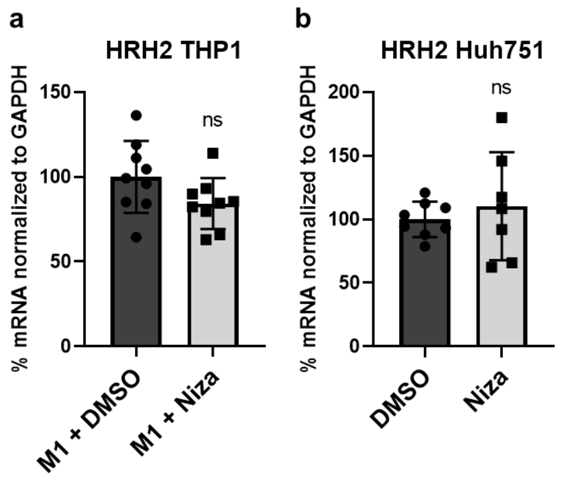


**Supplementary Figure 9: Absent effect of nizatidine on HRH2 expression in macrophage and Huh7.5.1 cell lines.** THP1-derived macrophages (a) or Huh7.5.1 cells (b) were treated with nizatidine for 3 days before assessment of HRH2 expression by qRT-PCR. (a) % mean + SD from 3 experiments performed in triplicate are shown (n=9); (b) % mean + SD from 2 experiments performed in quadruplicate are shown (n =8), 100% corresponding to non-treated cells as a reference. Ns = non-significant (unpaired t test). Source data are provided as a Source Data file.

# Supplementary Figure 10

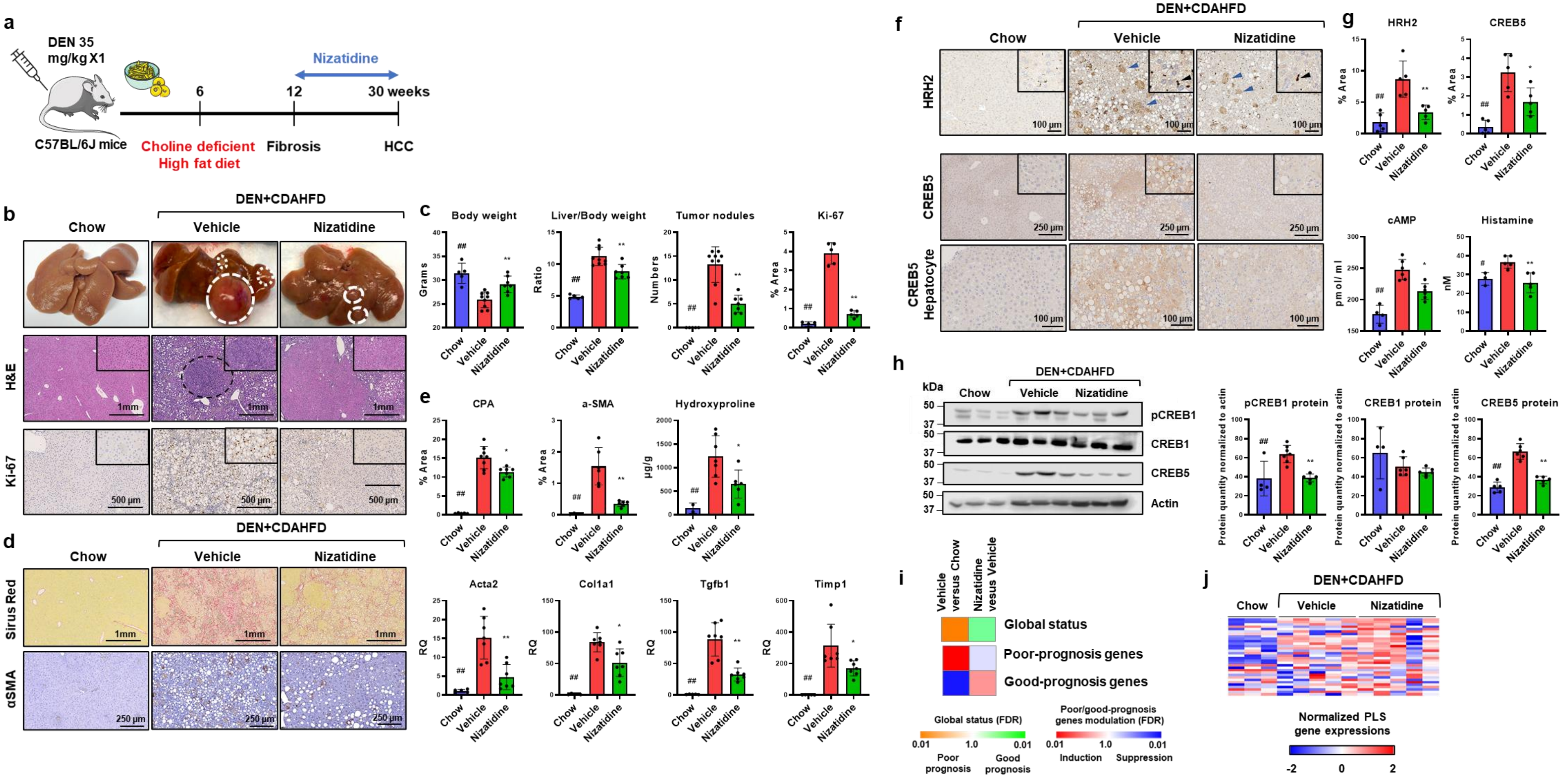

**Supplementary Figure 10: Nizatidine treatment robustly reduces liver fibrosis and carcinogenesis in a mouse model for NASH and HCC. a-c.** Nizatidine efficiently alleviates carcinogenesis. (a) Male C57Bl/6 mice received a single dose of DEN and were subjected to standard chow or choline-deficient, L-amino acid-defined, high-fat diet (CDAHFD). Chow died n = 5; CDAHFD + Vehicle n = 9; CDAHFD + Nizatidine n = 7. Animals received vehicle control or nizatidine for 18 weeks. (b) Representative morphometric analysis (hematoxylin & eosin (H&E)) of liver slices are shown (original magnification x5). White dotted line represents outlined tumor nodules. The number and size of macroscopic tumors were reported in (c). Surrogates used for tumor burden include liver/body weight ratio and surface nodules. Cell proliferation was assessed by Ki-67 staining. **d-e.** Nizatidine efficiently reduces liver fibrosis. Liver specimens were stained with Sirius red and fibrosis stage was evaluated through quantitative digital analysis of whole-scanned liver sections (collagen proportional area, CPA). αSMA staining, hydroxyproline quantification and fibrotic gene expression are shown. **f-g.** Expression of nizatidine target gene. Liver specimens were stained with anti-HRH2 or anti-CREB5 antibodies. Nuclei were counter stained. Original magnification x10 or x20 (for hepatocytes). Arrows show HRH2 positive macrophages. HRH2 and CREB5 expression was assessed by quantification of IHC. Tissue levels of cAMP and tissue histamine were measured by ELISA assay. **h.** Western blot analysis of phosphorylated (Ser 133) CREB1, total CREB1, and total CREB5. Beta-actin was used as a loading control. Quantification of western blot intensities (arbitrary units) was performed using image J software. **i.** PLS assessment in mouse liver tissue. Heatmaps show PLS global status (up) and PLS poor- and good-prognosis gene expression (bottom) **j.** Detailed PLS gene expression profiles. Heatmaps show the mean expression of the 32 gene signature (z scores of log2 normalized data). Gene expression was normalized according to 6 different housekeeping genes. All graphs show mean ± SD. # denotes $p<0.05$ and ## denotes $p<0.01$ comparing standard chow to DEN/CDAHFD. * denotes $p<0.05$ and ** denotes $p<0.01$ comparing DEN/CDAHFD + vehicle to DEN/CDAHFD + nizatidine (One-way ANOVA, followed by Tukey's multiple comparisons test). Source data are provided as a Source Data file.

**Supplementary Figure 11**

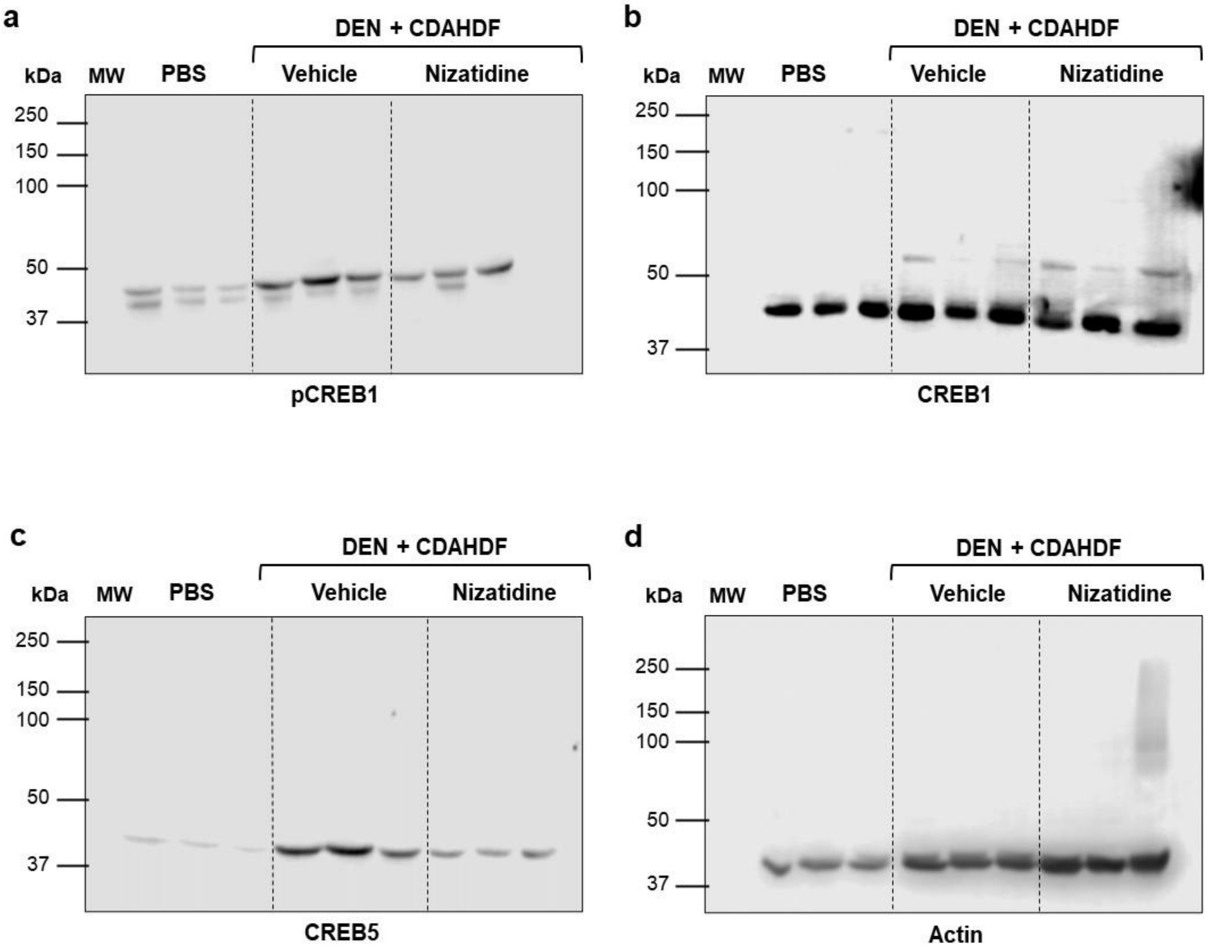


**Supplementary Figure 11 (related to Supplementary figure 10): Full length immunoblots from the DEN/CDAHDF mouse model.** To demonstrate the specificity of the applied antibodies, full-length gels are shown for Supplementary fig. 10. Target protein names indicate the bands shown in the Supplementary fig. 10. Protein analysis was performed in liver lysates from the same experiment using a reducing 12% SDS-PAGE gel electrophoresis. PVDF membranes were probed for pCREB1 and total CREB1 (rabbit monoclonal Ab, Cell Signaling, 1:1,000), CREB5 (mAb Ab, LS Bioscience, 1:1,000) and for Actin (mouse mAb, Abcam, 1:5,000). Due to the close molecular weight of the target proteins, analyses were performed on independent PVDF membranes done in the same conditions and using the same lysates. Secondary antibodies were horseradish peroxidase-conjugated goat anti-rabbit antibody (ThermoFisher Scientific 1:5,000) for pCREB1 and CREB1 and horseradish peroxidase-conjugated goat anti-mouse antibody (Cell Signaling, 1:1,5000) for CREB5 and Actin. The marker sizes (Precision Plus Protein Standards All Blue, BioRad) are indicated. Contrasts of western blotting images were equally adjusted for entire membranes using Bio-Rad image analysis software. MW = molecular weight markers. References of the antibodies are provided in Supplementary Table 4. Source data are provided as a Source Data file.

**Supplementary Figure 12**

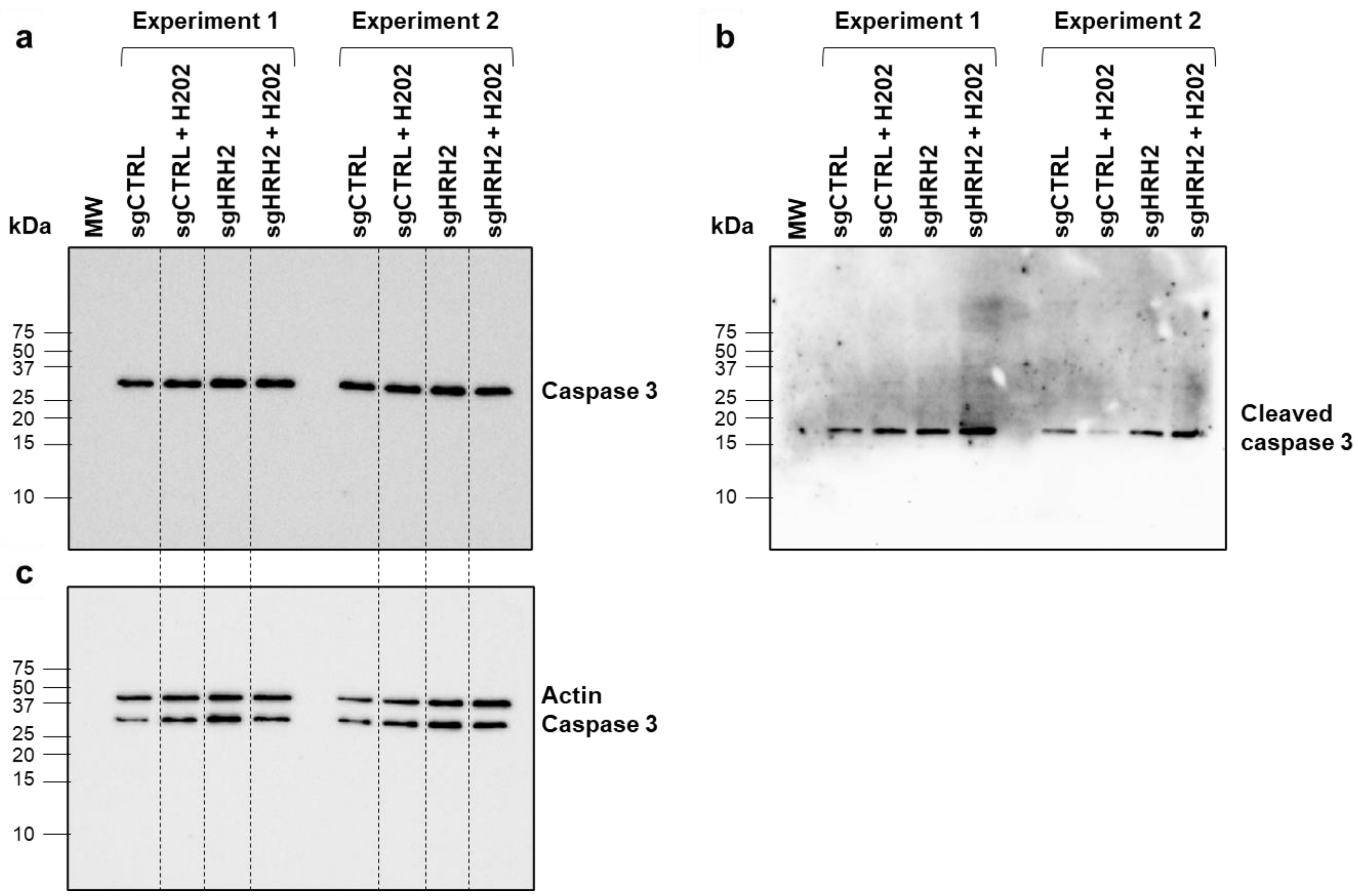


**Supplementary Figure 12 (related to Figure 5): Full length immunoblots from Figure 5.**
To demonstrate the specificity of the applied antibodies, full-length gels are shown for Fig. 5h. Target protein names indicate the bands shown in the Fig. 5h. Protein analysis was performed in cell lysates using a reducing 15% SDS-PAGE gel electrophoresis. **(a)** Analysis of total and **(b)** cleaved caspase 3 was performed on two independent PVDF membranes. **(c)** PVDF membrane were first probed for caspase 3 (Cell Signaling, 1:1,000) and then reprobed for Actin (Abcam, 1:2,000. Secondary antibody was horseradish peroxidase-conjugated goat anti-rabbit antibody (Jackson Immunoreasearch, 1:10,000) for caspase 3 and cleaved caspase 3 and horseradish peroxidase-conjugated goat anti-mouse antibody (GE HealthCare, 1:1,5000) for Actin. The marker sizes (Precision Plus Protein Standards All Blue, BioRad) are indicated. Contrasts of western blotting images were equally adjusted for entire membranes using Bio-Rad image analysis software. Two independent experiments are shown. Left panel (experiment 1) is presented in Fig. 5. MW = molecular weight markers. References of the antibodies are provided in Supplementary Table 4. Source data are provided as a Source Data file.

**Supplementary Figure 13**

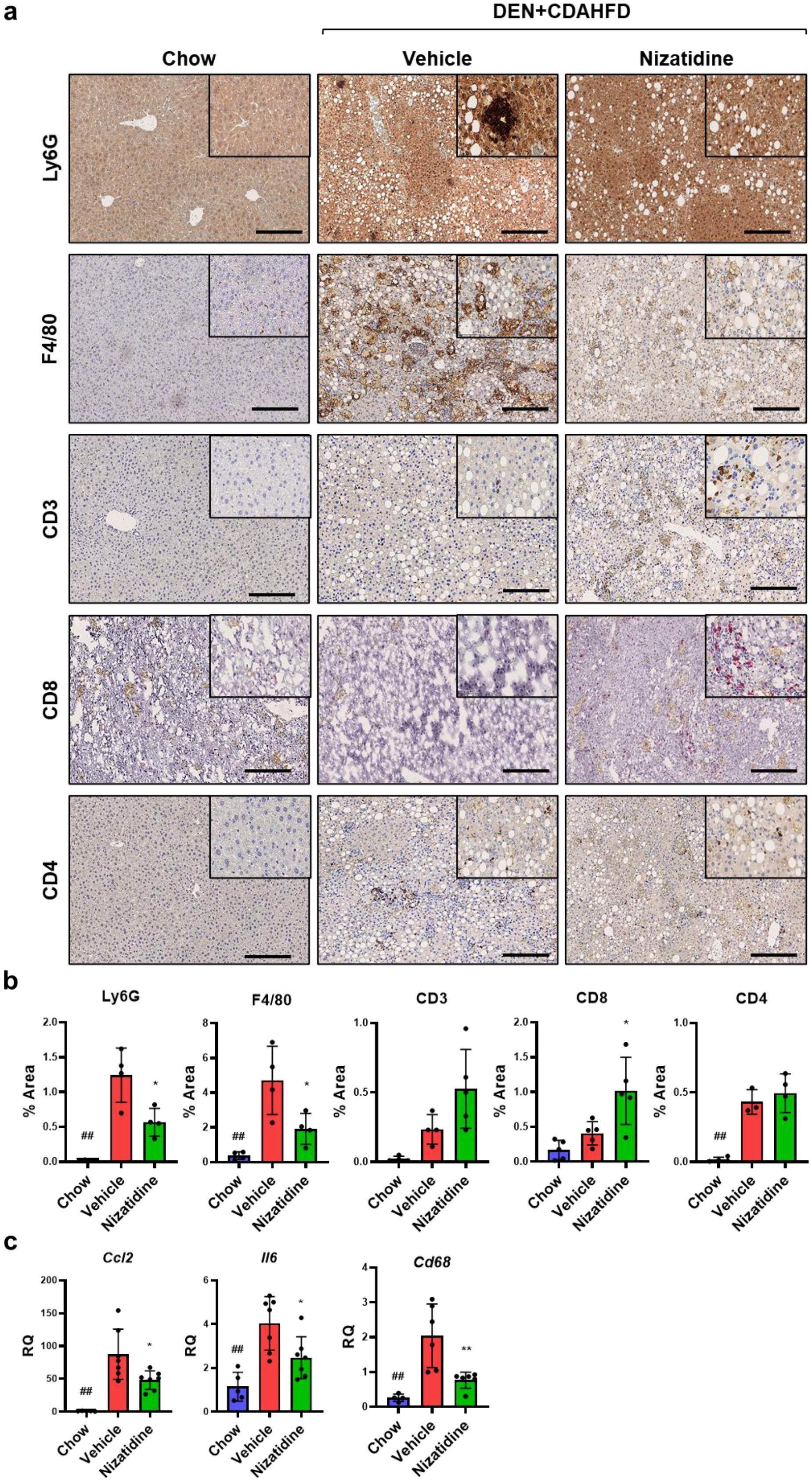

**Supplementary Figure 13 (related to Supplementary Figure 10: Immunohistochemical staining for immune cells. a.** Staining for lymphocyte antigen 6 complex locus G6D (Ly6G) (BD Biosciences), F4/80 (Linaris), cluster of differentiation 3 (CD3) (Zytomed Systems), cluster of differentiation 4 (CD4) (eBioscience), cluster of differentiation 8 (CD8) (BD Biosciences) in liver tissues of CTRL (chow), DEN/CDAHFD and DEN/CDAHFD nizatidine-treated mice are shown (one representative picture, original magnification x10; scale bar = 500 µm). **b.** Quantification of the different markers was performed using image J software. **c.** Gene expression (RT-qPCR) of pro-inflammatory cytokines *Ccl2* and *Il6* and cell marker *Cd68* are shown. All graphs show mean ± SD. # denotes $p<0.05$ and ## denotes $p<0.01$ comparing standard chow to DEN + CDAHFD. * denotes $p<0.05$ and ** denotes $p<0.01$ comparing DEN/CDAHFD + vehicle to DEN/CDAHFD + nizatidine (One-way ANOVA, followed by Tukey's multiple comparisons test). Source data are provided as a Source Data file.

## Supplementary Figure 14

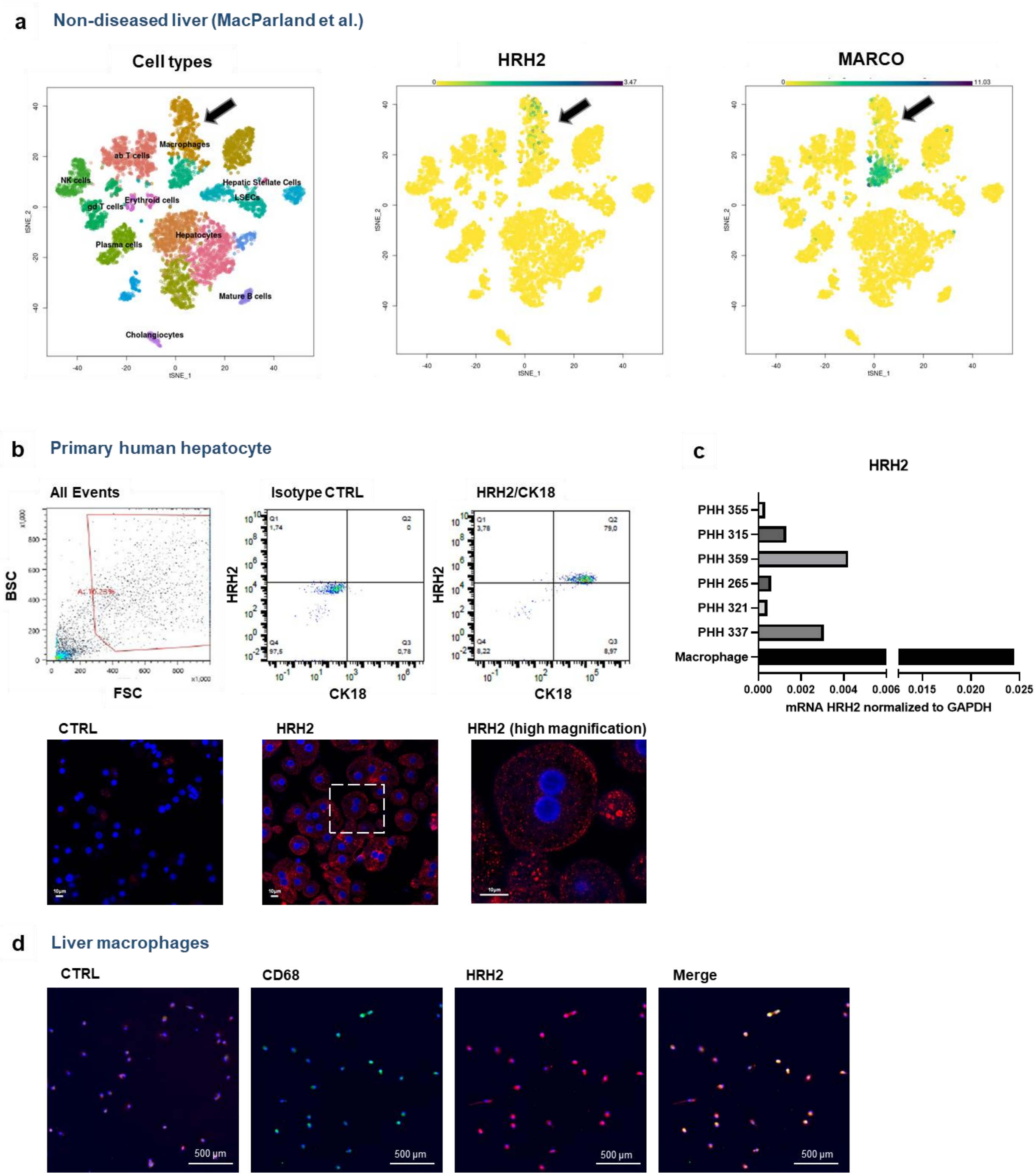

**Supplementary Figure 14 (related to Figure 6): HRH2 expression in non-diseased liver and isolated primary human hepatocyte (PHH) and macrophages. a.** t-SNE map of single-cell transcriptomes from normal liver tissue of donors without history of chronic liver disease highlighting the main liver cell compartments. Cells sharing similar transcriptome profiles are grouped by colors and each dot represents one cell. Arrows indicate macrophage compartment. Expression t-SNE map of HRH2 and MARCO are shown. Data are extracted from[3]. **b-d.** HRH2 expression in PHH and liver macrophages. (b) Upper panel: Immunodetection of HRH2 in PHH by flow cytometry. PHH freshly isolated from patient liver tissue were stained for HRH2 and CK18 (hepatocyte marker) or isotypes CTRL. Lower panel: Immunofluorescence staining of HRH2 in PHH in magenta (Alexa FluorTM 647) and nuclei in blue (DAPI) (confocal microscopy). (c) HRH2 mRNA expression in PHH from 6 patients by qRT-PCR compared with THP1-derived macrophages. Results are expressed as relative mRNA quantity normalized to GAPDH mRNA. (d) HRH2 expression in patient derived-liver macrophages. Macrophages were purified from liver tissue of patient without history of chronic liver disease by serial centrifugations and stained with anti-CD68 antibody (macrophage marker; FITC, green) and anti HRH2 antibody (Alexa FluorTM 647, magenta). Nuclei are counter stained in blue (DAPI) (epifluorescence microscopy). One experiment is shown. Source data are provided as a Source Data file.

**Supplementary Figure 15**

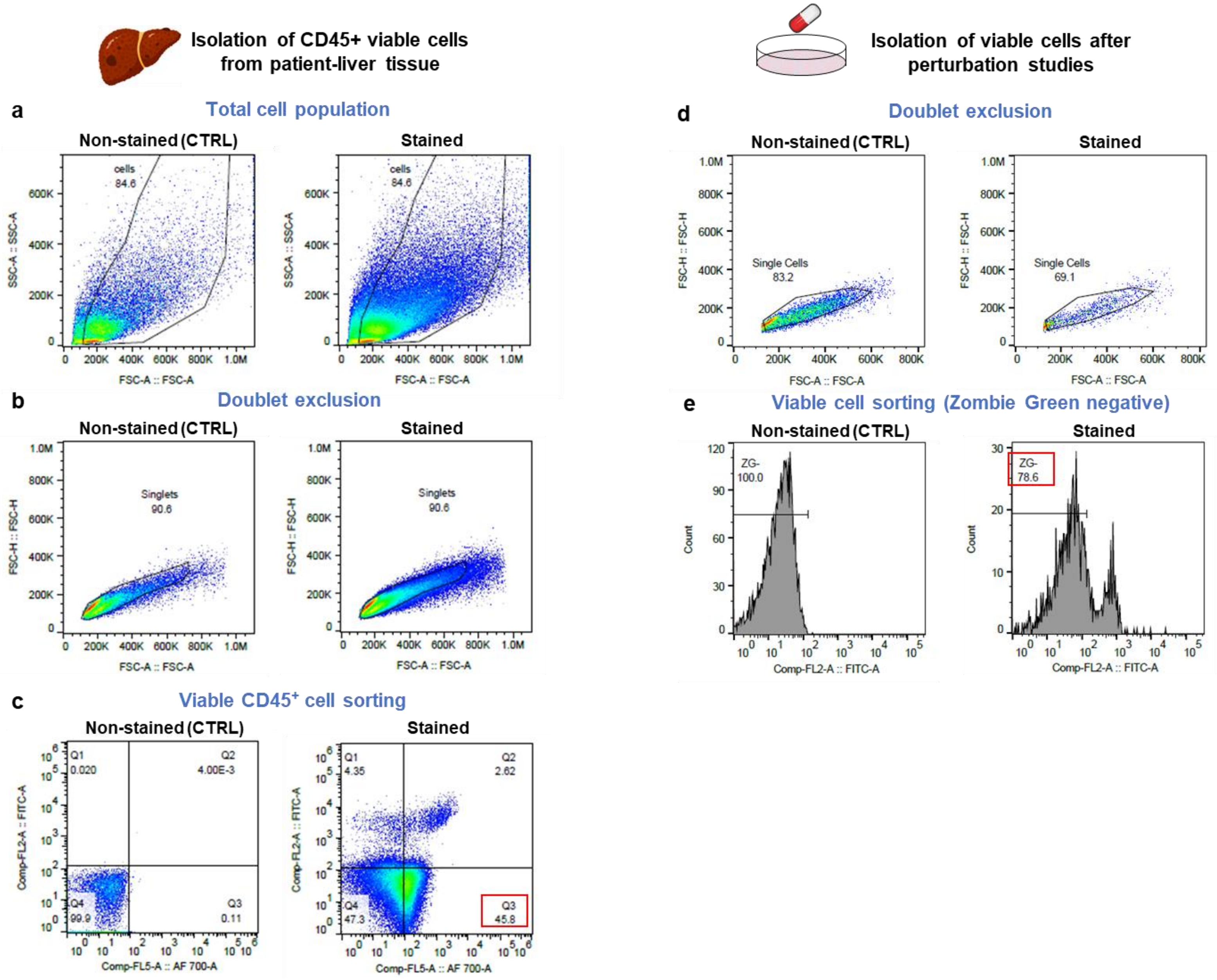

**Supplementary Figure 15 (related to Figure 6b). Flow cytometry gating strategy. a-c.** CD45+ leucocyte isolation from diseased patient-derived liver tissue by flow cytometry. After tissue dissociation, total cell population was stained using anti-CD45 antibody coupled with AF700 and zombie green (FITC) to detect viable cells. (a) The gating was performed on total cell population using FCS/SSC dot plots to remove cell debris. (b) The cells were then gated to isolate “singlets” and exclude “doublets” using plot through FSC-H and FSC-A parameters. (c) From the singlet gate, CD45 positive (AF700) and viable zombie green negative (FITC) cells were sorted. **d-e.** Gating strategy for the sorting of viable leucocyte isolated in a-c after perturbation studies. (d) The cells were gated to isolate “singlets”. (e) Viable cells (zombie green negative) were sorted in 384 well plates. Data were acquired using Sony SH800 Cell Sorter (Sony, Serial number: 0314067). Comp = compensation.

# Supplementary Figure 16

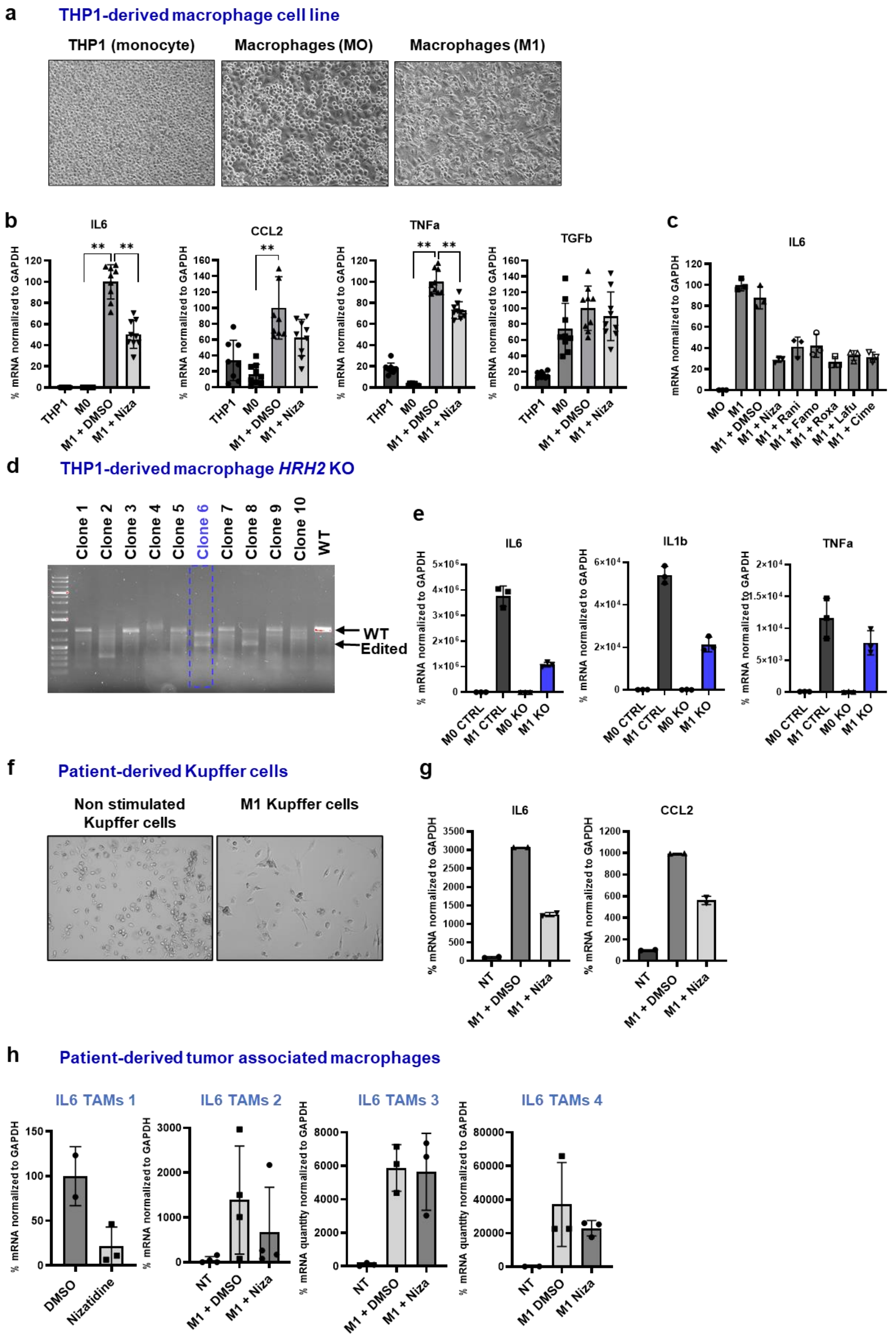

**Supplementary Figure 16 (related to Figure 6). Effect of nizatidine and *HRH2* KO on cytokine expression in a cell culture models for M1 macrophages. a.** Representative picture of differentiated macrophages. To generate macrophage-like cells (MO), THP-1 cells were treated with PMA. To generate M1-polarized THP-1 macrophages, THP-1 cells were treated with PMA plus LPS and IFNγ. M1-polarized THP-1 were treated with nizatidine (Niza) for 48 h. **b.** Gene expression of different cytokines was measured by qRT-PCR and normalized by GAPDH mRNA. Results are expressed as % mean + s.e.m from 3 independent experiments performed in triplicate (M1 + DMSO = 100%). * = $p < 0.05$; ** = $p < 0.01$; *** = $p < 0.001$ (two-tailed Mann-Whitney U test). **c.** Effect of HRH2 blockers on IL6 expression in M1-polarized macrophages (Rani = ranitidine; Famo = famotidine; Roxa = roxatidine; Lafu = lafutidine; Cime = cimetidine). Results are expressed as mean + SD and are representative of one out of three independent experiments performed in triplicate. **d-e.** *HRH2* KO perturbates cytokine expression in pro-inflammatory macrophages. (d) *HRH2* KO cells were generated using RNP technology (see method). Validation of sgRNA targeting HRH2 in THP1 cells after clonal selection. KO efficacy was assessed at genetic level by T7 endonuclease assay. Control cells correspond to parental THP1 cell line. Clone 6 was selected for further analysis. (e) Pro-inflammatory cytokines and markers expression were analyzed by qRT-PCR (mean +/- SD n= 3, one representative experiment out of two is shown). **f.** Representative images of M1 differentiated patient-derived Kupffer cells. To generate M1-polarized macrophages, patient Kupffer cells were treated with LPS and IFNγ. **g.** Gene expression of different cytokines was measured by qRT-PCR. Results are expressed as mean +/- SD from one experiment performed in duplicate. NT = non-treated (= 100%). **h.** IL6 expression in M1-polarized tumor associated macrophages (TAMs) isolated from different patient HCCs and treated with nizatidine. Results are expressed as mean +/- SD from one experiment performed in triplicate or quadruplicate. NT = non-treated (= 100%). Source data are provided as a Source Data file.

# Supplementary Figure 17

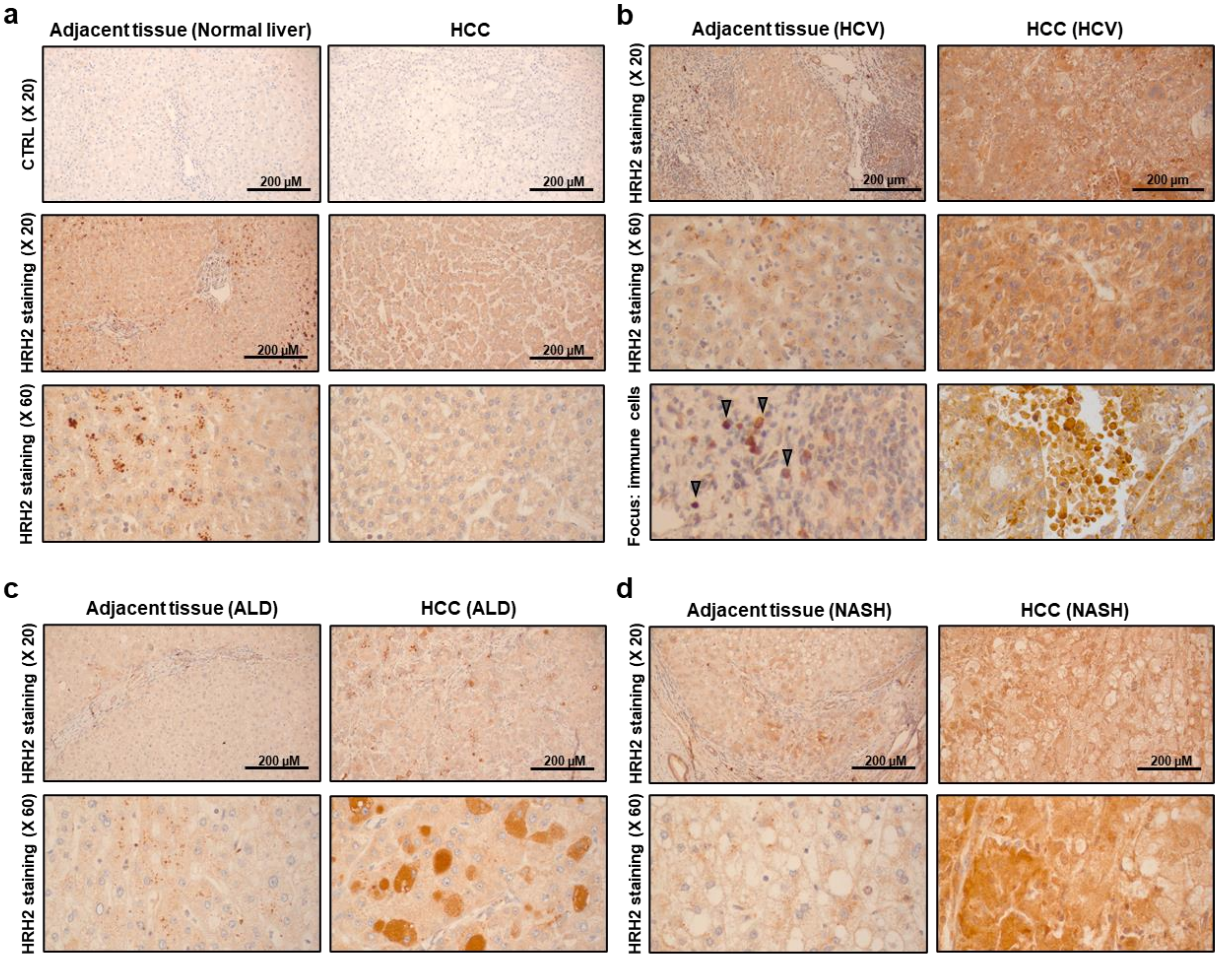


e

| Patient ID | HCC | Etiology of liver disease | Age at operation | Gender | IHC- score HCC | IHC-score liver |
|---|---|---|---|---|---|---|
| **1** | - | NAFLD, no fibrosis | 60 | female | - | 1 |
| **2** | - | NASH, portal and septal fibrosis | 60 | male | - | 1-2 |
| **3** | pT2, pN0 (0/3), M0, L0, V0, G1-G2, R0 | normal liver | 73 | male | 1-2 | 1 |
| **4** | rpT1, rpNx, L0, V0, G3, R0 | NAFLD, portal fibrosis, no cirrhosis | 56 | male | 2 | 1 |
| **5** | pT1b, pNx, L0, V0, Pn0, G2, R0 | NASH, portal and septal fibrosis, no cirrhosis | 73 | female | 3 | 1-2 |
| **6** | pT2, N0(0/2), L0, V0, G1, R0 | NASH, cirrhosis | 65 | male | 3 | 2-3 |
| **7** | pT2, pNx, L0, V1, G2, R0 | ASH-cirrhosis | 72 | male | 2 | 1-2 |
| **8** | pT1b, pN0(0/8), L0, V0, G2, R0 | HCV-cirrhosis | 70 | male | 2-3 | 1 |
| **9** | pT3, pN0 (0/2), L0, V1, G3, R0 | HCV, no cirrhosis | 72 | male | 2 | 1 |
| **10** | pT2, pNx, L0, V1, G3, R0 | HCV-cirrhosis | 56 | female | 3 | 1 |

**Supplementary Figure 17 (related to Figure 7): Representative pictures of HRH2 staining in liver tissues that were surgically resected from patients diagnosed with hepatocellular carcinoma (HCC).** For each patient, HCC and surrounding/adjacent liver tissue were stained. **a.** CTRL antibody. **a-d** HRH2 staining for HCC on normal liver plus negative control (a), chronic hepatitis C (HCV) with a focus on immune cells. Arrows indicate highly positive HRH2 immune cells. (b), alcoholic liver disease (ALD) (c), non-alcoholic steatohepatitis (NASH) (d). Magnification: 200x; focus: 600x. **e.** Table summarizing patient's characteristics and immunohistochemistry results. For each patient, HCC (if present) and surrounding liver tissue was stained. Images were taken with an Olympus BX45 microscope using Plan 20x objectives with a Gryphax Progress camera (Jenoptik) and Cell Sense software (Micro Optimal, Kirchheim / Teck): semiquantitative scoring of staining intensity was done from 0= negative, 1= faint staining, 2= moderate staining, to 3= strong staining intensity. TNM (Tumour, Node, Metastasis) and grading for HCC was done according to the current WHO-classification.

**Supplementary Table 1 (related to Figure 2): Drugs predicted to reverse the PLS using connectivity map (Cmap) analysis.** The *in-silico* screen was performed as described in Methods. Compounds with significant negative enrichment ($p$<0.05) are shown. For details about the metrics in the table, see www.broadinstitute.org/cmap. Enrichment score was calculated based on Kolmogorov–Smirnov statistic as previously reported[6] and $p$-value was calculated as nominal $p$-value based on random permutation of genes in the dataset (n=1,000). The score and $p$-value were used as measures to rank-order the compounds but not to set formal statistical significance, following statement from the American Statistical Association[7].

| Compound name | No. of instances | Enrichment score | *p*-value |
|---|---|---|---|
| CP-690334-01 | 8 | -0.620 | 0.002 |
| Genistein | 17 | -0.418 | 0.004 |
| Cefazolin | 5 | -0.708 | 0.005 |
| Pioglitazone | 11 | -0.500 | 0.005 |
| Bretylium tosylate | 4 | -0.770 | 0.006 |
| Triamcinolone | 5 | -0.692 | 0.006 |
| Isocarboxazid | 5 | -0.689 | 0.007 |
| Semustine | 4 | -0.746 | 0.008 |
| Melatonin | 4 | -0.745 | 0.008 |
| Primidone | 4 | -0.744 | 0.008 |
| 3-acetamidocoumarin | 4 | -0.740 | 0.009 |
| 2-aminobenzenesulfonamide | 4 | -0.738 | 0.009 |
| Bacampicillin | 4 | -0.717 | 0.013 |
| Resveratrol | 9 | -0.494 | 0.015 |
| Ethosuximide | 4 | -0.706 | 0.015 |
| Daunorubicin | 4 | -0.704 | 0.016 |
| Bacitracin | 3 | -0.792 | 0.018 |
| Chlorambucil | 4 | -0.694 | 0.019 |
| Methotrexate | 8 | -0.509 | 0.019 |
| Coralyne | 4 | -0.690 | 0.020 |
| Verapamil | 6 | -0.576 | 0.020 |
| SR-95531 | 4 | -0.684 | 0.022 |
| PNU-0251126 | 6 | -0.571 | 0.022 |
| Trimethobenzamide | 5 | -0.612 | 0.024 |
| Tetraethylenepentamine | 6 | -0.566 | 0.025 |
| Ramipril | 4 | -0.667 | 0.028 |
| PHA-00745360 | 8 | -0.488 | 0.028 |
| Oxetacaine | 5 | -0.592 | 0.033 |
| Captopril | 5 | -0.586 | 0.036 |
| Cyclic adenosine monophosphate | 4 | -0.647 | 0.037 |
| Demeclocycline | 6 | -0.539 | 0.038 |
| Nizatidine | 4 | -0.641 | 0.040 |
| Hydroxyachillin | 4 | -0.633 | 0.044 |
| Pralidoxime | 4 | -0.631 | 0.045 |
| Tolnaftate | 5 | -0.567 | 0.046 |
| Cefixime | 4 | -0.625 | 0.049 |

**Supplementary Table 2 (related to Figure 2): Drugs predicted to reverse the PLS in the Library of Integrated Network-based Cellular Signature (LINCS).** Compounds with significant connectivity score < - 90 are shown. For details about the metrics in the table, see www.lincscloud.org. The connectivity score (tau) is a standardized measure ranging from -100 to 100. For each perturbagen in the list of query results, the score corresponds to the fraction of reference gene sets with a greater similarity to the perturbagen than the current query. The reference gene sets are generated from all reference signatures of perturbagens in the CMap database (https://clue.io), and their connectivity to all other perturbagens have been precomputed as described in the portal (https://clue.io). A score of 95 indicates that only 5% of reference gene sets showed stronger connectivity than the current query to the perturbagen in question. It is important to note that because of redundancy within the reference panel, the score of 95 does not mean that 5% of activities exceed the query.

| Compound name | Connectivity score |
|---|---|
| KB-R7943 | -98,5833 |
| EX-527 | -98,1245 |
| Orteronel | -96,9669 |
| SB-202190 | -96,1775 |
| Isoliquiritigenin | -95,742 |
| U-0126 | -95,635 |
| Amlodipine | -95,5798 |
| Doxorubicin | -95,5556 |
| Mitomycin-c | -95,5199 |
| AS-605240 | -95,4162 |
| Etomoxir | -95,3171 |
| SJ-172550 | -95,2818 |
| Pimozide | -95,24 |
| Amiodarone | -95,1705 |
| PD-0325901 | -94,85 |
| Cediranib | -94,6909 |
| Penitrem-a | -94,6362 |
| Bisindolylmaleimide | -94,4585 |
| Serdemetan | -94,4503 |
| SN-38 | -94,4502 |
| Saracatinib | -94,4197 |
| RITA | -94,3435 |
| PD-0325901 | -94,3277 |
| L-733060 | -93,5492 |
| BRD-K64366758 | -93,4055 |
| PD-98059 | -93,2764 |
| RHO-kinase-inhibitor-III[rockout] | -93,2048 |
| RS-23597-190 | -92,8922 |
| Tivozanib | -92,8546 |
| AC-55649 | -92,8495 |
| Capsaicin | -92,6524 |
| BIBU-1361 | -92,5466 |
| Forskolin | -92,4131 |
| GW-501516 | -92,4021 |
| Rolipram | -92,3824 |
| Irinotecan | -92,3565 |
| BRD-K58247702 | -92,2239 |
| AS-703026 | -92,2207 |
| TG-101348 | -92,0564 |
| Brefeldin-a | -91,8994 |
| Selumetinib | -91,7074 |
| Lenalidomide | -91,5826 |
| GR-55562 | -91,5656 |
| Inhibitor-BEC | -91,1364 |
| Dexamethasone | -90,9814 |
| Daunorubicin | -90,8796 |
| Thiotepa | -90,8537 |
| Mycophenolic-acid | -90,6722 |
| MK-2206 | -90,6601 |
| Dactinomycin | -90,6236 |
| Promazine | -90,4945 |
| PD-184352 | -90,4244 |
| Clomifene | -90,1091 |
| Dilazep | -90,075 |

**Supplementary Table 3 (related to Figures 3 and 8):** Human liver tissues were obtained from patients undergoing liver resection with informed consent from all patients (see Methods). Table summarizes patient characteristics (age, gender, diagnosis, and treatments). CRLM = colorectal liver metastases; HCV = hepatitis C virus; NASH = non-alcoholic liver disease; ALD = alcoholic liver disease. Spheroids (Figure 3) were generated from liver tissue from patient without history of chronic liver disease. Tumorspheroids (Figure 8) were generated from HCC tissues from HCC patients.

| Donor | Etiology | Treatment | Disease stage |
|---|---|---|---|
| **Non-diseased** | | | |
| Patient 1 | CRLM | FOLFOX-AVASTIN | Healthy liver |
| Patient 2 | CRLM | FOLFOXIRI | Healthy liver |
| Patient 3 | CRLM | FOLFIRI | Healthy liver |
| Patient 4 | CRLM | FOLFOX-AVASTIN | Healthy liver |
| **HCC** | | | |
| HCC 1 | HCV | Harvoni | HCV + (1b) / A2F2 |
| HCC 2 | HCV | Ribavirine+Interferon | HCV+ (1a) / A0F2 |
| HCC 3 | Hereditary Hemochromatosis | / | Unknown |
| HCC 4 | NASH | / | F4 |
| HCC 5 | Unknown | / | No fibrosis / F0 |
| HCC 6 | ALD | / | Unknown |

**Supplementary Table 4 (related to Material and Method): Table of reagent and resource**

| REAGENT or RESOURCE | SOURCE | IDENTIFIER |
|---|---|---|
| **Antibodies** | | |
| Mouse monoclonal antibody anti-HBV surface antigen (HBsAg) | Leica Biosystems | NCL-HBsAG-2 clone 1044/341 |
| Alexa Fluor® 647 anti-mouse IgG (goat) | Jackson ImmunoResearch | Code: 115-605-003 |
| Alexa Fluor® 647 anti-human IgG (goat) | Jackson ImmunoResearch | Code: 109-605-003 |
| Alexa Fluor® 647 anti-rabbit IgG (donkey) | Invitrogen | Cat#A31573 |
| PE anti-Mouse IgG (H+L) Antibody, PE (goat) | Invitrogen | Cat#P-852 |
| Rabbit monoclonal anti-CREB1 (human) | Abcam | Cat#ab32515 |
| Rabbit monoclonal anti-pCREB1 (phosphoS133) (human) | Abcam | Cat#ab32096 |
| Mouse monoclonal anti-beta Actin-Loading CTRL (human) | Abcam | Cat#ab8226 |
| Mouse monoclonal anti-CREB5 | LsBio | Cat#LS-C62074-100 |
| Rabbit anti Caspase 3 | Cell Signaling | Cat# 9662S |
| Rabbit anti cleaved Caspase 3 | Cell Signaling | Cat# 9661S |
| Rabbit monoclonal antibody anti-CREB1 (rat and mouse) | Cell Signaling | Cat# 9197S |
| Rabbit monoclonal antibody anti-phospho CREB1 (rat and mouse) | Cell Signaling | Cat# 9198S |
| Mouse monoclonal anti-beta Actin-Loading CTRL (rat and mouse) | Abcam | Cat# ab8224 |
| Goat anti-mouse HRP | ThermoScientific | Cat#62-6520 |
| Goat anti-rabbit HRP | Cell Signaling | Cat#7074 |
| Goat anti-mouse HRP | GE HealthCare | Cat#NA931 |
| Goat anti-rabbit HRP | Jackson immunoresearch | Cat#111-035-144 |
| Rabbit IgG Isotype Control | Abcam | Cat#ab172730 |
| Mouse IgG Isotype Control | Invitrogen/ThermoFischer | Cat# 31903 |
| Rat anti-mouse F4/80 (clone BM8) | Linaris | Cat#T-2006 |
| Rat anti-mouse Ly-6G (clone 1A8) | BD Biosciences | Cat#BD 551459 |
| Rat anti-mouse CD8 (clone 53-6.7) | BD Biosciences | Cat#BD 553027 |
| Rabbit anti-mouse CD3 | Zytomed Systems | Cat#RBK024 |
| Rat anti-mouse CD4 | eBioscience | Cat#14-9766-82 |
| Rabbit polyclonal anti HRH2 | GeneTex | Cat#GTX108152 |
| Rabbit polyclonal anti mouse HRH2 | Abcam | Cat#ab215992 |
| Mouse monoclonal anti-HCV E2 (AP33) | Gift of Dr. A. Patel (MRC Virology Unit, Glasgow, UK) | N/A |
| Human IgG anti-HDV Ag | Purified from the serum of a HDV infected patient using MAbTrap Kit (GE Healthcare) | N/A |

| | | |
|---|---|---|
| Mouse monoclonal anti-DENV-E protein (clone 3H5-1) | Millipore | Cat#MAB8702 |
| Mouse Alexa Fluor® 700 anti-human CD45 Antibody | BioLegend | Cat#304024 |
| Peroxidase AffiniPure Goat Anti-Rabbit IgG | Jackson ImmunoReasearch | Cat#111-035-144 |
| Sheep Anti-mouse IgG, peroxidase-linked whole antibody | Amersham | Cat#NXA931 |
| Mouse monoclonal anti human CK18 | Agilent Dako | Cat# GA61861-2 |
| Mouse anti-HEV ORF2 | Millipore | Cat#AB-827236 |
| **Chemicals, Peptides, and Recombinant Proteins** | | |
| Absolute ethanol | VWR Chemicals | Cat#20821330 |
| Erlotinib | LC Laboratories | Cat#E-4007 |
| IFNα-2a | Sigma-Aldrich | Cat#SRP4594 |
| Pioglitazone | Sigma-Aldrich | Cat#E6910 |
| Primidone | Sigma-Aldrich | Cat#P7295 |
| Triamcinolone | Sigma-Aldrich | Cat#T6376 |
| Pimozide | Sigma-Aldrich | Cat#P1793 |
| Dilazep HCl2 | Sigma-Aldrich | Cat#D5294 |
| Dorzolamide | Sigma-Aldrich | Cat#SML0468 |
| DMSO | Sigma-Aldrich | Cat#41640 |
| Oleic acid | Sigma-Aldrich | Cat#O1008 |
| Palmitic acid | Sigma-Aldrich | Cat#P0500 |
| Recombinant EGF | Sigma-Aldrich | Cat#E9644 |
| Fr180204 | Sigma-Aldrich | Cat#SML0320 |
| Oxetacaine | Santa Cruz | Cat#sc-253222 |
| Resveratrol | Santa-Cruz | Cat#sc-200808 |
| Pralidoxime | Santa Cruz | Cat#sc-212578 |
| Captopril | Selleckchem | Cat#S2051 |
| Selumetinib | Selleckchem | Cat#S1008 |
| MK-2206 | Selleckchem | Cat#S1078 |
| Nizatidine | Selleckchem | Cat#S1890 |
| Famotidine | Selleckchem | Cat#S2078 |
| Ranitidine hydrochloride | Selleckchem | Cat#S1801 |
| Roxatidine hydrochloride | Selleckchem | Cat#S1880 |
| Lafutidine | Selleckchem | Cat#S2065 |
| Cimetidine | Selleckchem | Cat#S1845 |
| Clomifene citrate | Selleckchem | Cat#S2561 |
| PD-0325901 | Selleckchem | Cat#S1036 |
| Pimasertib | Selleckchem | Cat#S1475 |
| Tivozanib | Selleckchem | Cat#S1207 |
| Brefeldin-a | Selleckchem | Cat#S7046 |
| Rolipram | Selleckchem | Cat#S1430 |
| TG-101348 | Selleckchem | Cat#S2736 |
| CI-10140 | Selleckchem | Cat#S1020 |

| | | |
|---|---|---|
| Isoliquiritigenin | Selleckchem | Cat#S2404 |
| Tolnaftate | Selleckchem | Cat#S2058 |
| Cediranib | Selleckchem | Cat#S1017 |
| Orteronel | Selleckchem | Cat#S1195 |
| Tipifarnib | Selleckchem | Cat#S1453 |
| Histamine | Selleckchem | Cat#S4118 |
| H89 | Selleckchem | Cat#S1582 |
| Daclatasvir | Selleckchem | Cat#S1482 |
| Sofosbuvir | Selleckchem | Cat#S2794 |
| 8-CPT cAMP | Abcam | Cat#ab120424 |
| Clarity WB ECL reagent | Bio-rad | Cat#170-5061 |
| DAPI | Life Technologies | Cat#D1306 |
| DEN | Sigma-Aldrich | Cat#N0756 |
| FLAG peptide | Sigma-Aldrich | Cat#F3290 |
| Anti-FLAG M2 affinity gel | Sigma-Aldrich | Cat#A2220 |
| TRIzol | ThermoFisher scientific | Cat#15596026 |
| iScript™ RT-qPCR Sample Preparation Reagent | Bio-rad | Cat#1708898 |
| Collagenase from Clostridium histolyticum | Sigma | Cat#C5138 |
| Zombie green | Biolegend | Cat#423111 |
| Trypan blue | Sigma | Cat#T8154 |
| Lipofectamine RNAiMax | ThermoFisher | Cat #13778150 |
| Lipopolysaccharides from Escherichia col | Sigma-Aldrich | Cat#L4391 |
| Recombinant Human IFN-gamma Protein | R&D systems | Cat#285-IF-100 |
| Percoll® | Sigma-Aldrich | Cat#P1644 |
| Corning® BioCoat™ Cellware, Collagen Type I, Corning | Corning | Cat#354444 |
| **Critical Commercial Assays** | | |
| cAMP Parameter Assay Kit | R&D Systems | Cat#KGE002B |
| Histamine ELISA kit | Abcam | Cat#ab213975 |
| Direct-zol RNA MiniPrep | Zymo Research | Cat#R2060 |
| ReliaPrep RNA Cell Miniprep System | Promega | Cat#Z6012 |
| SuperScript™ III First-Strand Synthesis System | Invitrogen | Cat#18080051 |
| Pierce BCA Protein Assay Kit | ThermoFisher | Cat#23227 |
| CalPhos™ Mammalian Transfection Kit | Clontech Laboratories | Cat#631312 |
| MammoCult™ Human Medium Kit | Stemcell™ Technologies | Cat#05620 |
| Heparin | Stemcell™ Technologies | Cat#07980 |
| Hydrocortison | Stemcell™ Technologies | Cat#74142 |
| Fungizone | InvivoGen | Cat code: ant-fn-1 |
| Primocin | InvivoGen | Cat code: ant-pm-05 |
| CellTiter-Glo® 3D Cell Viability Assay | Promega | Cat#G9681 |

| | | |
|---|---|---|
| Click-iT EdU Flow Cytometry Cell Proliferation Assay | ThermoFisher | Cat# C10420 |
| Tumor Dissociation Kit, human | Miltenyi Biotec | Cat#130-095-929 |
| **Oligonucleotides** | | |
| SMART-compatible primer for HCV RNA amplification: 5'-Biotin-AAGCAGTGGTATCAACGCAGAGT ACTCTGCGGAACCGGTGAGTA-3' | Sigma-Aldrich | N/A |
| sgRNA eGFP (CTRL): GGTGAACCGCATCGAGCTGA | Broad Institute | N/A |
| sgRNA CREB5.1: GGCTTGCTGAATCACAACGT | Broad Institute | N/A |
| sgRNA CREB5.2: GTTCTTCAGGAATCTCGTTG | Broad Institute | N/A |
| sgRNA CREB5.3: AATGGGAACATGAACACCAT | Broad Institute | N/A |
| sgRNA HRH2: ATCCATGAACCTGGCTTCG | Broad Institute | N/A |
| sgHrh2 (HTVI): ATTGAAAGTCACCATCAGTG | Chopchop V3 | N/A |
| sgCTRL non-targeting (HTVI): GTGAACCGCATCGAGCTGA | Chopchop V3 | N/A |
| sgTp53: GACCCTGTCACCGAGACCCC | Chopchop V3 | N/A |
| Human IL6 TaqMan® Gene Expression Assays | ThermoFisher | Cat# Hs00174131_m1 |
| Human IL1b TaqMan® Gene Expression Assays | ThermoFisher | Cat# Hs01555410_m1 |
| Human TNFa TaqMan® Gene Expression Assays | ThermoFisher | Cat# Hs00174128_m1 |
| Human HRH2 TaqMan® Gene Expression Assays | ThermoFisher | Cat# Hs00254569_s1 |
| Human<br>CCL2 fw: 5’-AGCCACCTTCATTCCCCAAG-3’<br>CCL2 rev: 5’-TCTCCTTGGCCACAATGGTC-3’ | Sigma-Aldrich | Primer-blast, https://www.ncbi.nlm.nih.gov/tools/primer-blast/index.cgi?LINK_LOC=BlastHom) |
| Human<br>TGFb fw; 5′-TACCTGAACCCGTGTTGCTCTC-3′<br>TGFb rev: 5′-GTTGCTGAGGTATCGCCAGGAA-3′ | Sigma-Aldrich | Sequence from [8] |
| Mouse Acta2 | ThermoFisher | Cat# Mm00725412_s1 |
| Mouse Col1a1 | ThermoFisher | Cat# Mm00801666_g1 |
| Ccl2 | ThermoFisher | Cat# Mm00441242_m1 |
| Cd68 | ThermoFisher | Cat# Mm03047343_m1 |
| Il6 | ThermoFisher | Cat# Mm00446190_m1 |
| Timp1 | ThermoFisher | Cat# Mm01341361_m1 |

| | | |
|---|---|---|
| Tgfb1 | ThermoFisher | Cat# Mm01178820_m1 |
| Silencer® Select HRH2 siRNA | Ambion (ThermoFisher) | Cat# s6911 |
| Silencer® Select Negative Control #1 siRNA | Ambion (ThermoFisher) | Cat# 4390843 |
| **Recombinant DNA** | | |
| pFK-J6-JFH1 chimaera (HCV Jc1) | Plasmid for production of HCVcc Jc1 is a gift of Dr R. Bartenschlager | N/A |
| pXPR_BRD111 Cas9 vector | Addgene | Cat#78166 |
| pMD2.G envelope vector | Addgene | Cat#12259 |
| psPAX2 lentiviral vector | Addgene | Cat#12260 |
| pXPR_BRD016 sgRNA expression vector | Broad Institute | N/A |
| CRISPR in vitro vector pX459 | Plasmid is a gift of Dr Feng Zhang | Addgene plasmid # 62988 |
| CRISPR in vivo vector pX330 | Plasmid is a gift of Dr Feng Zhang | Addgene plasmid # 42230 |
| CMV-SB13 | Plasmid is a gift of Dr. Darjus Tschaharganeh | N/A |
| pT3-EF1α-KRASG12D-IRES-EGFP | Dr. Darjus Tschaharganeh | N/A |

## Supplementary Method

***DMSO-differentiation time course experiment (Supplementary Fig. 1).*** RNA-Seq was performed on DMSO-differentiated Huh7.5.1 cell line on day 0, 3, 7 and 10 in triplicates. The raw RNA-Seq reads were aligned to the hg19 reference using the spliced gap aligner STAR2.7, with count-based quantitation carried out via the Subread package feature Countsat the gene levels for annotation builds. The raw read counts on genes were further normalized across samples to obtain the log CPM values using edge Rby assuming the negative binomial distribution. The day 0 (without DMSO) was used as internal control to compare with cells harvested at day 3-10 (under DMSO) to obtain the gene expression fold change, which is further followed by the Gene Set enrichment Analysis (GSEA).

***Similarity of global transcriptome between the Huh7.5.1$^{dif}$ cells and single-cell transcriptome profiles of liver cells isolated from human cirrhotic livers (Supplementary Fig. 5)***. Single cell RNA-Seq data of 62,210 cells from both cirrhotic and healthy livers were obtained from[2]. "Seurat" was used to integrate single cell datasets, normalize gene expression, and unsupervised cluster cells. The cell types were grouped in clusters: T cell (19560), Mononuclear Phagocytes (10942), Innate Lymphoid cell (12788), Endothelia (7857), Epithelia (5122), Mesenchyme (2716), B Cell (1946), Plasma Cells (859), Plasmacytoid Dendritic Cell (331), and Mast Cells (89). Mononuclear Phagocytes were further divided in eight subtypes. For each cell types, the log2 fc of gene expression between cirrhotic and healthy liver cells were calculated. In parallel, the log2 fc of gene expression were calculated between HCV-infected Huh7.5.1$^{dif}$ cells vs Mock-infected Huh7.5.1$^{dif}$ cells (Bulk microarray). To assess the similarity of differential expression between bulk and each cell type, the Spearman correlation was calculated between the log2fc of gene expression from bulk data scRNA-Seq data, followed by the signed -log10 p values, indicating both direction and significance of the similarity. For PLS deconvolution, the scRNA-Seq data set from healthy livers was extracted from GSE115469[3] Mean gene expression levels of PLS members were calculated in each cell type.

***PLS gene expression in HCV-infected Huh7.5.1$^{dif}$ cells and similarity of HCV-infected Huh7.5.1$^{dif}$ cells with different liver cell compartment at the single cell level (Supplementary Fig. 5).*** Read were aligned using hisat2 v2.0.4. Gene reads for hg19 and JC1 HCV genomes were counted using htseq-count. Counts were normalized using DESeq2 and further processed using Seurat v4.0.2. C8 msigDB cell type gene set enrichments for cells were calculated using GSVA v1.38.2 or similar.

***M1 macrophage polarization, Kupffer cell and TAM isolation (Supplementary Fig. 16)***. M1 macrophage polarization from THP-1 was done as described[8]. Kupffer cells and Tumor associated macrophages (TAMs) were isolated as described[9] from liver tissue of patients without history of chronic liver disease and from HCC tumor tissue respectively. Cells then were treated with nizatidine 20 µM or DMSO vehicle for 48 h.

**Supplementary references:**


1. Hoshida, Y. *et al.* Gene expression in fixed tissues and outcome in hepatocellular carcinoma. *N. Engl. J. Med.* **359**, 1995–2004 (2008).
2. Ramachandran, P. *et al.* Resolving the fibrotic niche of human liver cirrhosis at single-cell level. *Nature* (2019) doi:10.1038/s41586-019-1631-3.
3. MacParland, S. A. *et al.* Single cell RNA sequencing of human liver reveals distinct intrahepatic macrophage populations. *Nat. Commun.* **9**, 4383 (2018).
4. Nakagawa, S. *et al.* Molecular liver cancer prevention in cirrhosis by organ transcriptome analysis and lysophosphatidic acid pathway inhibition. *Cancer Cell* **30**, 879–890 (2016).
5. King, L. Y. *et al.* A genomic and clinical prognostic index for hepatitis C-related early-stage cirrhosis that predicts clinical deterioration. *Gut* **64**, 1296 (2015).
6. Subramanian, A. *et al.* Gene set enrichment analysis: A knowledge-based approach for interpreting genome-wide expression profiles. *Proc. Natl. Acad. Sci.* **102**, 15545–15550 (2005).
7. Wasserstein, R. L. & Lazar, N. A. The ASA statement on p-values: context, process, and purpose. *Am. Stat.* **70**, 129–133 (2016).
8. Yeung, O. W. H. *et al.* Alternatively activated (M2) macrophages promote tumour growth and invasiveness in hepatocellular carcinoma. *J. Hepatol.* **62**, 607–616 (2015).

9. Kegel, V. *et al.* Protocol for isolation of primary human hepatocytes and corresponding major populations of non-parenchymal liver cells. *J. Vis. Exp. JoVE* e53069 (2016) doi:10.3791/53069.
