# Supplementary material for: A human liver cell-based system modeling a clinical prognostic liver signature for therapeutic discovery
Source: Nat Commun. 2021 Sep 17;12:5525. doi: 10.1038/s41467-021-25468-9 (PMC8448834; doi:10.1038/s41467-021-25468-9)
Supplement: Supplementary file 3 — Description of Additional Supplementary Files [file 41467_2021_25468_MOESM3_ESM.pdf]

**Description of Additional Supplementary Files**

File Name: Supplementary Data 1
Description: List of the 73 poor-prognosis genes and of the 113 good-prognosis genes of the PLS1. The reduced version of the PLS corresponds to 32 genes bioinformatically selected and validated in patient cohort4,5. These 32 genes are highlighted in blue. The 6 housekeeping genes used to normalize the PLS gene expression are also listed.

File Name: Supplementary Data 2
Description: Gene expression profiles of uninfected (Control) and HCV Jc1-infected Huh7.5.1dif cells were analyzed by GSEA as described in Methods. The molecular pathway gene sets were retrieved from Molecular Signature Database (MSigDB, www.broadinstitute.org/msigdb). Gene sets with significant enrichment (FDR <0.25) or top 20 are shown. NES: normalized enrichment score, FDR: false discovery rate.
