# Supplementary material for: A human liver cell-based system modeling a clinical prognostic liver signature for therapeutic discovery
Source: Nat Commun. 2021 Sep 17;12:5525. doi: 10.1038/s41467-021-25468-9 (PMC8448834; doi:10.1038/s41467-021-25468-9)
Supplement: Supplementary file 6 — Reporting Summary [file 41467_2021_25468_MOESM6_ESM.pdf]

nature research

Corresponding author(s): Prof. Thomas Baumert

Last updated by author(s): 07/22/2021

Reporting Summary

Nature Research wishes to improve the reproducibility of the work that we publish. This form provides structure for consistency and transparency in reporting. For further information on Nature Research policies, see Authors & Referees and the Editorial Policy Checklist .

Statistics

For all statistical analyses, confirm that the following items are present in the figure legend, table legend, main text, or Methods section.

n/a	Confirmed	
☐	☒	The exact sample size (*n*) for each experimental group/condition, given as a discrete number and unit of measurement
☐	☒	A statement on whether measurements were taken from distinct samples or whether the same sample was measured repeatedly
☐	☒	The statistical test(s) used AND whether they are one- or two-sided<br>*Only common tests should be described solely by name; describe more complex techniques in the Methods section.*
☒	☐	A description of all covariates tested
☐	☒	A description of any assumptions or corrections, such as tests of normality and adjustment for multiple comparisons
☐	☒	A full description of the statistical parameters including central tendency (e.g. means) or other basic estimates (e.g. regression coefficient) AND variation (e.g. standard deviation) or associated estimates of uncertainty (e.g. confidence intervals)
☐	☒	For null hypothesis testing, the test statistic (e.g. *F*, *t*, *r*) with confidence intervals, effect sizes, degrees of freedom and *P* value noted<br>*Give P values as exact values whenever suitable.*
☒	☐	For Bayesian analysis, information on the choice of priors and Markov chain Monte Carlo settings
☒	☐	For hierarchical and complex designs, identification of the appropriate level for tests and full reporting of outcomes
☐	☒	Estimates of effect sizes (e.g. Cohen's *d*, Pearson's *r*), indicating how they were calculated

Our web collection on statistics for biologists contains articles on many of the points above.

Software and code

Policy information about availability of computer code

Data collection	None
Data analysis	GraphPad PRISM 8 for Windows; Image Lab (Bio-Rad, version 5.2.1), Gene Set Enrichment Analysis (GSEA) 4.0.3, implemented in GenePattern genomic analysis toolkits, Morpheus, STAR v2.7. , MSigDB ver.4.0, TopHat v2.1, Cufflinks v2.2, cmap (www.broadinstitute.org/cmap) and LINCS database (https://clue.io/), GENE-E (www.broadinstitute.org/GENE-E), Image J IHC ToolBox, Cell Sense, R version 3.5.3 with package "RaceID" and HISAT, CytExpert 2.3 software and FlowJo V10.5.3. SH800 cell sorter software V2.1.5, Seurat v4.0.2.

For manuscripts utilizing custom algorithms or software that are central to the research but not yet described in published literature, software must be made available to editors/reviewers. We strongly encourage code deposition in a community repository (e.g. GitHub). See the Nature Research guidelines for submitting code & software for further information.

Data

Policy information about availability of data

All manuscripts must include a data availability statement. This statement should provide the following information, where applicable:

- Accession codes, unique identifiers, or web links for publicly available datasets
- A list of figures that have associated raw data
- A description of any restrictions on data availability

All data generated in this study are provided in the Supplementary materials, in Source data file or have been deposited at NCBI Gene Expression Omnibus database. For specific questions, contact the corresponding authors and lead contact, Prof. Thomas F. Baumert (thomas.baumert@unistra.fr).
The genomic dataset generated in this study and presented in Fig. 1, Fig. 2, Fig. 4g-h, Fig. 6 and Fig. 8a as well as Supplementary Figs. 1 and 3 have been deposited at NCBI Gene Expression Omnibus database as a super series under accession code GSE66843 (https://www.ncbi.nlm.nih.gov/geo/query/acc.cgi?acc=GSE66843) “A cell-based model unravels drivers for hepatocarcinogenesis and targets for clinical chemoprevention”. Sub-series:
- GSE66841 (https://www.ncbi.nlm.nih.gov/geo/query/acc.cgi?acc=GSE66841). Gene expression profiles of Huh7.5.1 and HepG2 cells infected with HCV, HBV, HDV,

and alcohol, and clinical liver tissues treated with various compounds of co-culture with LX2 cells.
- GSE66842 (https://www.ncbi.nlm.nih.gov/geo/query/acc.cgi?acc=GSE66841). Gene expression profiles of differentiated Huh7.5.1 cells infected with HCV Jc1 clone.
- GSE81040 (https://www.ncbi.nlm.nih.gov/geo/query/acc.cgi?acc=GSE81040). High-throughput single-cell RNA-Seq profiling of DMSO-differentiated Huh7.5.1 undergoing or not long-term HCV infection.
- GSE81801 (https://www.ncbi.nlm.nih.gov/geo/query/acc.cgi?acc=GSE81801). Prognostic Liver Signature profiles of Jc1-infected Huh7.5.1dif cells treated with various drugs.
- GSE115473 (https://www.ncbi.nlm.nih.gov/geo/query/acc.cgi?acc=GSE115473). Transcriptome profiles of liver from cirrhotic rat treated with nizatidine.
- GSE173671 (https://www.ncbi.nlm.nih.gov/geo/query/acc.cgi?acc=GSE173671). A human liver cell-based system modeling a clinical prognostic liver signature combined with single-cell RNA-Seq for discovery of liver disease therapeutics.
- GSE169084 (https://www.ncbi.nlm.nih.gov/geo/query/acc.cgi?acc=GSE169084). Bulk RNA sequencing of DMSO differentiated Huh751 at different time points
The 186 and 32 PLS gene lists and the full 186 gene signature heatmaps are provided in Supplementary Data 1 and Supplementary Fig 2. Full immunoblots are provided in Supplementary Figs 7, 8, 11 and 12. Results of the transcriptome-based in silico drug screening using the PLS as a query in the chemogenomic database connectivity map and LINCS are available within Supplementary information (Tables 1 and 2). The source data underlying the Figs. 3, 4, 5, 8 and Supplementary Figs. 9, 10, 13, and 16 are presented as a Source Data File. The following public databases were used in the study are available on https://www.ncbi.nlm.nih.gov/geo/query:
- GSE14520 (https://www.ncbi.nlm.nih.gov/geo/query/acc.cgi?acc=GSE14520). HBV-related liver cancer patient cohort
- GSE28619 (https://www.ncbi.nlm.nih.gov/geo/query/acc.cgi?acc=GSE28619). Alcoholic hepatitis patient cohort
- GSE49541 (https://www.ncbi.nlm.nih.gov/geo/query/acc.cgi?acc=GSE49541) NASH patient cohort
- GSE54102 (https://www.ncbi.nlm.nih.gov/geo/query/acc.cgi?acc=GSE54102). HCV-related cirrhosis, US
- GSE15654 (https://www.ncbi.nlm.nih.gov/geo/query/acc.cgi?acc=GSE15654). HCV-related cirrhosis, Italy
- GSE31803 (https://www.ncbi.nlm.nih.gov/geo/query/acc.cgi?acc=GSE31803) NAFLD patient cohort
- GSE48452 (https://www.ncbi.nlm.nih.gov/geo/query/acc.cgi?acc=GSE48452). NASH patient cohort
- GSE115469 (https://www.ncbi.nlm.nih.gov/geo/query/acc.cgi?acc=GSE115469). Human liver cellular landscape by single cell RNA-seq
- GSE124395 (https://www.ncbi.nlm.nih.gov/geo/query/acc.cgi?acc=GSE124395). Human liver cell atlas
- GSE136103 (https://www.ncbi.nlm.nih.gov/geo/query/acc.cgi?acc=GSE136103) Human liver cirrhosis using single-cell transcriptomics
- GSE84346 (https://www.ncbi.nlm.nih.gov/geo/query/acc.cgi?acc=GSE84346). HCV infected patient cohort
- GSE48452 (https://www.ncbi.nlm.nih.gov/geo/query/acc.cgi?acc=GSE48452). Obesity, NAFLD, NASH patient cohort
- GSE10143 (https://www.ncbi.nlm.nih.gov/geo/query/acc.cgi?acc=GSE10143). Cirrhosis patient cohort
- GSE94660 (https://www.ncbi.nlm.nih.gov/geo/query/acc.cgi?acc=GSE94660). HBV-HCC patient cohort
- GSE94399 (https://www.ncbi.nlm.nih.gov/geo/query/acc.cgi?acc=GSE94399). Alcoholic hepatitis patient cohort

Field-specific reporting

Please select the one below that is the best fit for your research. If you are not sure, read the appropriate sections before making your selection.

- [x] Life sciences
- [ ] Behavioural & social sciences
- [ ] Ecological, evolutionary & environmental sciences

For a reference copy of the document with all sections, see nature.com/documents/nr-reporting-summary-flat.pdf

Life sciences study design

All studies must disclose on these points even when the disclosure is negative.

Sample size

- For in vivo experiments, sample size was determined using a power calculation. We reasoned that a 50% reduction in tumor incidence would be a meaningful finding based on past studies examining HCC chemoprevention strategies in animal models. Therefore, our sample size estimate was based on a p-value of 0.01 at 90% power assuming a 50% difference in means in tumor burden with 33% standard deviation between control and drug-treated animals.
- Clinical data in this manuscript were retrospectively explored in terms of the associations with gene expressions/modulations. Therefore, sample size calculations were not performed.
- For in vitro experiments, no sample-size calculation was performed. Individual experiments were reproduced three times in an independent manner with similar results. The precise number (n) of biologically independent samples used to derive statistics is indicated in the figure legends.

Data exclusions

- In the rat DEN model, two of the DEN animals that received vehicle control died. These animals were excluded from analysis due to lack of viable tissue and blood available for analysis.
- For HTVI, one animal was excluded for technical reason (no efficient HTVI)
- For cell culture/in vitro data, poor or insufficient technical quality of experiment or data analysis resulted in exclusion of samples (empirical method: Excluded values if X < or > to $\mu \pm \sigma$)
- Cinical data were publicly available from Gene Expression Omnibus (GEO) (https://www.ncbi.nlm.nih.gov/) and used according to the attached annotations. There were no excluded data.

Replication

Individual experiments were reproduced at least three in an independent manner with similar results except otherwise stated. The exact sample size is indicated in each figure legend.

Randomization

Animals were randomly assigned to receive vehicle control or nizatidine. Not relevant for in vitro study.

Blinding

Investigators were blinded to group allocation during data collection of gross tumor counts. Histological staining and immunohistochemistry was performed blinded and pathological assessment and morphometric quantification of the staining was blinded. Liver function tests were

performed blinded.

Reporting for specific materials, systems and methods

We require information from authors about some types of materials, experimental systems and methods used in many studies. Here, indicate whether each material, system or method listed is relevant to your study. If you are not sure if a list item applies to your research, read the appropriate section before selecting a response.

Materials & experimental systems

n/a	Involved in the study
☐	☒ Antibodies
☐	☒ Eukaryotic cell lines
☒	☐ Palaeontology
☐	☒ Animals and other organisms
☐	☒ Human research participants
☒	☐ Clinical data

Methods

n/a	Involved in the study
☒	☐ ChIP-seq
☐	☒ Flow cytometry
☒	☐ MRI-based neuroimaging

Antibodies

Antibodies used

The list of antibodies used in this study is provided in Supplementary Table 4. The dilution for western blot anlayses is specified in each figure legend.

Validation

Anti-HDV IgG was purified from serum of a patient infected with hepatitis B and D viruses and validated by our laboratory as described Verrier et al., Hepatology 2016;63:35-48 and Verrier et al., Gut 2020;69:158-167.
All commercially available antibodies had been validated by the manufacturers and are widely used in the scientific community for Western blotting. Detectable band at the correct molecular weight and observation of a response to stimuli confirmed the validity of the antibody. Full-length immunoblots are provided in the Supplementary figures 7, 8 and 11, 12.

Eukaryotic cell lines

Policy information about cell lines

Cell line source(s)

Huh7.5.1 (Human hepatocarcinoma-derived cell line) are a gift of Dr. F. Chisari (The Scripps Research Institute, La Jolla, CA). HepG2 (Human hepatocarcinoma-derived cell line) and THP1 (Human monocyte-derived cell line) were purchased from ATCC. LX2 (Human Hepatic Stellate Cell Line) were purchased from Merck. For each cell line, the culture conditions and the corresponding references (characterization) are indicated in Materials and Methods section.

Authentication

Huh7.5.1 cells were used at low passage number and regularly monitored for their ability to support HCV infection. The cell lines used were not authenticated.

Mycoplasma contamination

All the cell lines were mycoplasma negative (mycoplasma contamination is routinely controlled every 50 days using PlasmoTest™ - Mycoplasma Detection Kit, InvivoGen rep-pt1)

Commonly misidentified lines
(See ICLAC register)

No commonly misidentified cell lines were used

Animals and other organisms

Policy information about studies involving animals; ARRIVE guidelines recommended for reporting animal research

Laboratory animals

Eight-week old male Wistar rats (Charles River Laboratories, Wilmington, Massachusetts) and two-week old male C57Bl/6 mice (Charles River Laboratories, Wilmington, MA). For HTVI, C57BL/6 male mice were purchased at an age of 8-weeks old from Janvier Labs were used in this study. Details about experimental procedure are provided in Materials and Methods section ("Research experiments on live vertebrates").

Wild animals

The study did not involved wild animals

Field-collected samples

The study did not involved samples collected from the field

Ethics oversight

For rat and mouse models for liver disease and HCC, animals were housed in accordance with the guidelines of the Massachusetts General Hospital Institutional Animal Care and Use Committee (protocol approval numbers 2007N000113 and 2009N000207) and received human care according to the criteria outlined in the "Guide for the Care and Use of Laboratory Animals" of the National Academy of Sciences.
For HTVI, aAll experiments were performed in accordance with the German law and the governmental bodies, and with approval from the the Regierungspräsidium Karlsruhe providing the ethical oversight of the study (approval number G39/18). Mice were housed at the German Cancer Research Center (DKFZ) (constant temperature of 20-24°C and 45-65% humidity with a 12-h light-dark cycle) and maintained under specific pathogen-free conditions.

Note that full information on the approval of the study protocol must also be provided in the manuscript.

Human research participants

Policy information about studies involving human research participants

Population characteristics	Human liver tissues were obtained from liver disease patients undergoing liver resection with informed consent from all patients for de-identified use at the Center for Digestive and Liver Disease of the Strasbourg University Hospitals University of Strasbourg, France (DC-2016-2616 and RIPH2 LivMod IDRCB 2019-A00738-49, ClinicalTrial NCT04690972) or at Mount Sinai Hospital, New York City, NY (HS13-00159). A brief summary of patient characteristics (diagnosis and treatments) is provided in Supplementary Table 3.
Recruitment	Not relevant for this study. Due to the rarity of the patient liver tissues, we did not exclude patient.
Ethics oversight	Human liver tissues were obtained from liver disease patients undergoing liver resection with informed consent from all patients for de-identified use at the Center for Digestive and Liver Disease of the Strasbourg University Hospitals University of Strasbourg, France (DC-2016-2616 and RIPH2 LivMod IDRCB 2019-A00738-49, ClinicalTrial NCT04690972) or at Mount Sinai Hospital, New York City, NY (HS13-00159). The protocols were approved by the local Ethics Committee of the University of Strasbourg Hospitals and Mount Sinai Hospital, respectively. All material was collected during a medical procedure strictly performed within the frame of the medical treatment of the patient. Informed consent is provided according to the Declaration of Helsinki. Detailed patient information and informed consent procedures are implemented by the Strasbourg University Hospital Biological Resources Center (HUS CRB). Patients were given an information sheet which outlines that their left-over biological material (liver resection and blood samples) that was collected during their medical treatment is requested for research purposes. All patients received and signed an informed consent form in order to provide authorization or refuse the use of their biological samples (protocols DC-2016-2616 and RIPH2 LivMod IDRCB 2019-A00738-49 ClinicalTrial NCT04690972). The patients maintain the right to withdraw their consent at any time and to request the destruction of their biological material which is strictly respected. While there was clinical descriptive data available, the identity of the patients was protected by internal coding. A brief summary of patient characteristics (diagnosis and treatments) is provided in Supplementary Table 3.<br>Formalin-fixed, paraffin-embedded human tissue samples were provided by the tissue biobank of the University Medicine Mainz in accordance to the ethics committee of the Medical Association of the State of Rhineland Palatinate, Germany (Immunhistochemical analysis of HCC, No. 837.146.17 (10980), April 24th, 2017).

Note that full information on the approval of the study protocol must also be provided in the manuscript.

Flow Cytometry

Plots

Confirm that:

- [x] The axis labels state the marker and fluorochrome used (e.g. CD4-FITC).
- [x] The axis scales are clearly visible. Include numbers along axes only for bottom left plot of group (a 'group' is an analysis of identical markers).
- [x] All plots are contour plots with outliers or pseudocolor plots.
- [x] A numerical value for number of cells or percentage (with statistics) is provided.

Methodology

Sample preparation	Sample preparation is described in Material and Method section "Edu assay and Flow cytometry" and "Single-cell RNA-Seq analyses of patient liver tissues"
Instrument	Cell sorting was performed on Sony SH800 Cell Sorter (Sony, Serial number: 0314067). Flow cytometry data were acquired using Cytoflex B2R2U0 cytometer (Beckman Coulter, BA47394
Software	Data were acquired using Sony SH800 Cell Sorter (Sony, Serial number: 0314067) and SH800 cell sorter software V2.1.5 then analyzed using FlowJo V10.5.3 or F using Cytoflex B2R2U0 cytometer (Beckman Coulter, BA47394) and CytExpert 2.3 software and then analyzed using FlowJo V10.5.3.
Cell population abundance	A total of 150,000 cells were analyzed and 56,000 CD45+ cells were sorted. A post-sort control were performed by an analysis of the post-sort fraction.
Gating strategy	The gating was first performed using a FCS/SSC dot plot. The gain used was fixed when all populations was observed on the plot. The main population was gated to perform a singlets plot using FSC-H and FSC-A parameters. The histogram plot and the dot plots were created from the singlets gate using Count/APC or FITC or SSC-A/APC or FITC parameters. Gating strategy for cell sorting is presented in Supplementary figure 13.

- [x] Tick this box to confirm that a figure exemplifying the gating strategy is provided in the Supplementary Information.
